# Identification of Novel MicroRNAs and Their Diagnostic and Prognostic Significance in Oral Cancer

**DOI:** 10.3390/cancers11050610

**Published:** 2019-04-30

**Authors:** Luca Falzone, Gabriella Lupo, Giusy Rita Maria La Rosa, Salvatore Crimi, Carmelina Daniela Anfuso, Rossella Salemi, Ernesto Rapisarda, Massimo Libra, Saverio Candido

**Affiliations:** 1 Department of Biomedical and Biotechnological Sciences, Oncologic, Clinic and General Pathology Section, University of Catania, 95123 Catania, Italy; luca.falzone@unict.it (L.F.); lupogab@unict.it (G.L.); anfudan@unict.it (C.D.A.); rossellasalemi@alice.it (R.S.); scandido@unict.it (S.C.); 2 Research Center for Prevention, Diagnosis and Treatment of Cancer, University of Catania, 95123 Catania, Italy; 3 Department of General Surgery and Surgical-Medical Specialties, University of Catania, 95125 Catania, Italy; g_larosa92@live.it (G.R.M.L.R.); errapis@tin.it (E.R.); 4 Department of Surgical and Biomedical Sciences, University of Catania, 95123 Catania, Italy; torecrimi@gmail.com

**Keywords:** oral cancer, miRNA, bioinformatics, datasets, biomarkers, TCGA, GEO DataSets

## Abstract

*Background*: Oral cancer is one of the most prevalent cancers worldwide. Despite that the oral cavity is easily accessible for clinical examinations, oral cancers are often not promptly diagnosed. Furthermore, to date no effective biomarkers are available for oral cancer. Therefore, there is an urgent need to identify novel biomarkers able to improve both diagnostic and prognostic strategies. In this context, the development of innovative high-throughput technologies for molecular and epigenetics analyses has generated a huge amount of data that may be used for the identification of new cancer biomarkers. *Methods*: In the present study, GEO DataSets and TCGA miRNA profiling datasets were analyzed in order to identify miRNAs with diagnostic and prognostic significance. Furthermore, several computational approaches were adopted to establish the functional roles of these miRNAs. *Results*: The analysis of datasets allowed for the identification of 11 miRNAs with a potential diagnostic role for oral cancer. Additionally, eight miRNAs associated with patients’ prognosis were also identified; six miRNAs predictive of patients’ overall survival (OS) and one, hsa-miR-let.7i-3p, associated with tumor recurrence. *Conclusions*: The integrated analysis of different miRNA expression datasets allows for the identification of a set of miRNAs that, after validation, may be used for the early detection of oral cancers.

## 1. Introduction

Oral cancer is one of the most prevalent cancers worldwide, accounting for about 354,864 new diagnoses and approximately 177,384 new deaths annually [1]. Generally, the term oral cancer identifies a subset of head and neck cancers arising in the lips, hard palate, upper and lower alveolar ridges, anterior two-thirds of the tongue, sublingual region, buccal mucosa, retro-molar trigone and floor of the mouth [2]. Among these cancers, the most frequent histotype is oral squamous cell carcinoma (OSCC) representing about 95% of all oral cancers [3]. Recent epidemiological data demonstrated that despite the development of novel screening strategies together with the advancement of pharmacological treatments, the incidence and mortality rates of head and neck cancer, and in particular that of oral cancer, are almost stable or increased during the last years [4,5].

Behind the increase of both oral cancer incidence and mortality rates, there are several modifiable factors, including dietary and lifestyles habits, together contributing to cancer development. Among these factors, alcohol consumption and smoking represent the most recognized factors predisposing to OSCC [6,7]. Additionally, viruses and other microbes have been intensively associated with a higher increase of OSCC development, such as infections sustained by human papilloma viruses (HPVs), Epstein-Barr virus (EBV) or Candida albicans [8,9,10]. Although the majority of the studies are focused on the investigation of microbial factors as cancer risk factors, recently several studies were pursued with the aim of establishing a potential role of the human microbiota in protecting the host from several tumors, including those of the oral cavity [11,12,13].

Along with these well-recognized risk factors, oral cancer development is also associated with several molecular alterations affecting key genes involved in the regulation of pivotal cellular processes, such as cell cycle, cell proliferation and apoptosis. The most frequent gene alterations found linked with OSCC affect *TP53*, *NOTCH1*, *CDKN2A*, *SYNE1*, *PIK3CA*, as well as the EGFR pathway-related genes (including *TGF-β*, fibroblastic growth factor-BP (*FGF-BP*) and *MMK6*) [14,15]. Recently, epigenetic modifications, including promoter/intragenic methylation and microRNAs (miRNAs) de-regulation, have been linked to the development of oral cancers by mediating the alteration of cellular homeostasis and physiological processes [16,17,18].

Despite that the oral cavity is readily explorable, most oral tumors are diagnosed at an advanced stage reducing the survival rate of patients [19,20]. Currently, there are no effective biomarkers for the early diagnosis of oral cancer. Several studies have proposed the evaluation of the salivary and serum levels of IL-6 and/or IL-8 as promising biomarkers for oral cancer lesions, however, the sensitivity and specificity of these markers were low because they increase also in presence of various oral cavity inflammatory conditions [21,22]. Other studies focused the attention on tumor markers already used for the diagnosis of other solid tumors, such as the salivary levels of the carcino-embryonic antigen (CEA; 68.9% sensitivity, 73.3% specificity) [23], carcinoantigen 19-9 (CA19-9; no diagnostic value) [24] and CA125 (80.0% sensitivity, 66.0% specificity) [25]. However, the sensitivity and specificity of these markers were not high enough to diagnose effectively all oral tumors.

Therefore, there is an urgent need to identify novel biomarkers for the early diagnosis of oral cancer. In this context, the role of non-coding RNAs, of which miRNAs are the most studied, has been recently acquiring remarkable importance in the development of several pathologies, including cancer [26,27,28]. In particular, several studies demonstrated that miRNAs, a class of small non-coding RNAs with a length of 20–22 nucleotides, are involved in cancer, including that of oral cavity cancer, inducing epigenetic modifications altering key cellular processes, such as cell differentiation, growth, apoptosis and drug resistance [29,30]. Notably, miRNAs are able to regulate gene expression by controlling mRNA translation, either by translational repression of the targeted mRNA or by enhancing its degradation through an RNA interference mechanism [31]. Furthermore, a growing body of evidence demonstrated that dysregulated miRNAs may be used for diagnostic and prognostic purposes. In fact, it is well established that certain miRNAs are specifically associated with the presence of tumors, even in the early stages, or associated with a worse prognosis [32,33].

Therefore, miRNAs may represent good candidate biomarkers also for oral cancer. On this matter, during the last decade, a huge amount of molecular and bioinformatics data has been generated, with the final goal of characterizing miRNAs’ expression profile in several cancers. These databases were therefore used to identify new effective biomarkers identified through computational approaches [34]. Several studies analyzed the data deriving from miRNAs microarray or sequencing profiling in oral cancer samples. However, the lack of integration between the different data matrix generated has generated confusing data on this matter. For instance, Manikandan M and colleagues (2016) have performed a miRNA microarray analysis in a discovery cohort (*n* = 29) and validation cohort (*n* = 61) of primary OSCC tissue specimens identifying a set of miRNAs (let-7a, let-7d, let-7f, miR-16, miR-29b, miR-142-3p, miR-144, miR-203, miR-223 and miR-1275) potentially involved in oral cancer development and progression [35]. Other microarray studies have identified miRNAs different from those identified by Manikandan et al. In particular, Chamorro Petronacci and colleagues (2019) have recently identified two potential miRNAs, miR-497-5p and miR-4417 associated with the presence of OSCC [36]. Yan ZY and co-workers (2017) have identified seven key miRNAs (miR-21, miR-31, miR-338, miR-125b, hsa-miR-133a, miR-133b and miR-139) associated with the tumor [37]. Therefore, it is evident that there are no concordant data generated by the single and independent analysis of different miRNA microarray datasets for oral cancer.

To our best knowledge, no previous studies have analyzed simultaneously different oral cancer tissue miRNAs profiling datasets. In the present study, miRNA expression datasets, contained in both the Gene Expression Omnibus DataSets (GEO DataSets) and The Cancer Genome Atlas (TCGA) Head and Neck Cancer (HNSC), were analyzed to identify a panel of miRNAs used as potential diagnostic and/or prognostic biomarkers for oral cancer.

## 2. Results

### 2.1. Identification of Oral Cancer-Associated miRNAs

The differential analysis performed by GEO2R on the two datasets of the GEO DataSets database allowed the identification of two lists of de-regulated miRNAs in oral tumors compared to non-tumor controls. By comparing these two lists of miRNAs, it was possible to identify 28 miRNAs differentially expressed in the tumor tissue, 12 of which were up-regulated and 16 were down-regulated (Table 1).

The analysis of the expression data of miRNAs contained in the TCGA HNSC dataset allowed us to obtain a list of 514 de-regulated miRNAs associated with the presence of a tumor (*p* < 0.01; Table S1). Furthermore, 21 of the 28 miRNAs identified with the GEO DataSets analysis were contained in this list of 514 miRNAs (Table S1), thus confirming that the results obtained from the two analyses were overlapping.

To further narrow the search towards miRNAs showing a strong diagnostic significance, the 25 most up-regulated and the 25 most down-regulated miRNAs were selected from the list of 514 miRNAs. The analysis of the TCGA HNSC dataset showed a list of 50 miRNAs that were strongly associated with the presence of the tumor (Table 2).

In Table 2, in bold, are reported the miRNAs matching between the analyses of GEO DataSets and TCGA datasets. These common-shared miRNAs are presumably more involved in neoplastic transformation mechanisms underlying the development of oral cancers. As shown in Table 2, most of these miRNAs presented the highest levels of up-regulation (miR-196a-5p and miR-196b-5p) and down-regulation (miR-99a-5p, miR-133a-3p, miR-1-3p and miR-375-3p).

In summary, the two differential analyses between tumor samples and normal samples performed on GEO DataSets and TCGA datasets, showed that 11 miRNAs, of which four up-regulated and seven down-regulated, were strictly related to the presence of a tumor (Table 3).

As shown in Table 3, the miRNA miR-196a-5p and the two miRNAs miR-1-3p and miR-375-3p, respectively up-regulated and down-regulated, presented the higher levels of over-expression or down-regulation in all three datasets (two GEO DataSets and one TCGA).

For the further prediction analyses of target genes and altered molecular pathways, the 11 miRNAs reported in Table 3 were considered: hsa-miR-196a-5p, hsa-miR-196b-5p, hsa-miR-503-5p, hsa-miR-18a-5p, hsa-miR-379-5p, hsa-miR-195-5p, hsa-miR-411-5p, hsa-miR-99a-5p, hsa-miR-133a-3p, hsa-miR- 1-3p and hsa-miR-375-3p.

### 2.2. Levels of Interaction Between the 11 Selected miRNAs and Oral Cancer Altered Genes

Through the use of COSMIC and mirDIP, the majority of mutated and altered genes in oral cavity tumors were identified and miRNA-gene interaction specificity was determined, respectively. First, by using COSMIC the 10 most frequent mutations and gene alteration found in oral cancers were identified. These altered genes were the *TP53* genes (43%), *FAT1* (28%), *CASP8* (23%), *TERT* (22%), *NOTCH1* (20%), *CDKN2A* (16%), *HRAS* (10%), *KMT2D* (10%), *FGFR3* (8%) and *PIK3CA* (8%).

**Figure 1:**
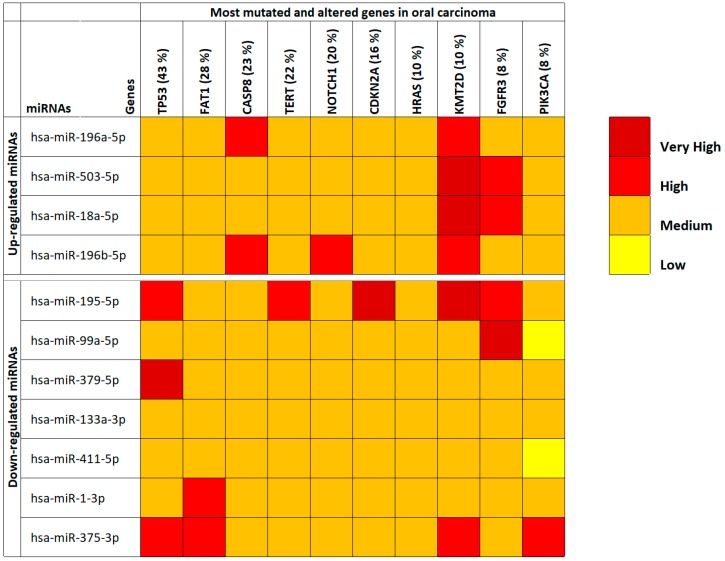
mirDIP analysis of interaction levels between selected miRNAs and the main mutated and altered genes in oral cavity tumors.

Then, through mirDIP it was possible to establish the interaction levels with the selected 11 oral cancer-associated miRNAs and the genes identified by using COSMIC (Table S2). For the 10 interacting genes, also gene expression levels were analyzed using the TCGA HNSC IlluminaHiSeq pancan normalized dataset (Table S3). This analysis revealed that all the identified miRNAs were able to target the commonly mutated genes in oral cancers. In fact, the majority of the interactions occurred with medium-high specificity underlining the strong correlation between deregulated miRNAs in cancer patients and the aforementioned genes involved in fundamental cellular and cancer pathways (Figure 1). However, the analysis of the TCGA HNSC IlluminaHiSeq pancan normalized dataset showed that only six out of the 10 (*TP53*, *FAT1*, *CASP8*, *TERT*, *CDKN2A* and *PIK3CA*) genes were significantly de-regulated in oral cancers (Table S3).

The most interesting data showed in Figure 1 were relative to the *KMT2D* gene where it is possible to note how all up-regulated miRNAs were able to target this gene by reducing its expression levels. This is important if we consider that KMT2D is a tumor suppressor gene, therefore its down-regulation due to the suppressive action of up-regulated miRNAs triggers cellular neoplastic transformation. Taking into account the miRNAs, it can instead be noted that, generally, the 11 selected miRNAs have medium levels of interaction with the target genes (medium interaction orange). However, the down-regulated hsa-miR-195-5p and hsa-miR-375-3p miRNAs showed the highest interaction levels with the analyzed genes (Figure 1). The expression levels of the 10 targeted genes showed that the *FAT1*, *CASP8*, *TERT*, *CDKN2A* and *PIK3CA* genes were significantly up-regulated in tumor samples, while *TP53* was significantly down-regulated.

### 2.3. Correlation Analysis Between the 11 Selected Tumor-Associated miRNAs and Ene Expression

The correlation value of each miRNA with different genes was obtained by using the bioinformatics tool miRCancerdb. This tool is a free easy-to-use database of microRNA-gene/protein expression and correlation in cancer where the correlation levels are calculated using the Pearson correlation coefficient (ρ). Therefore, the correlation levels are denoted by “*r*” [38].

**Figure 2:**
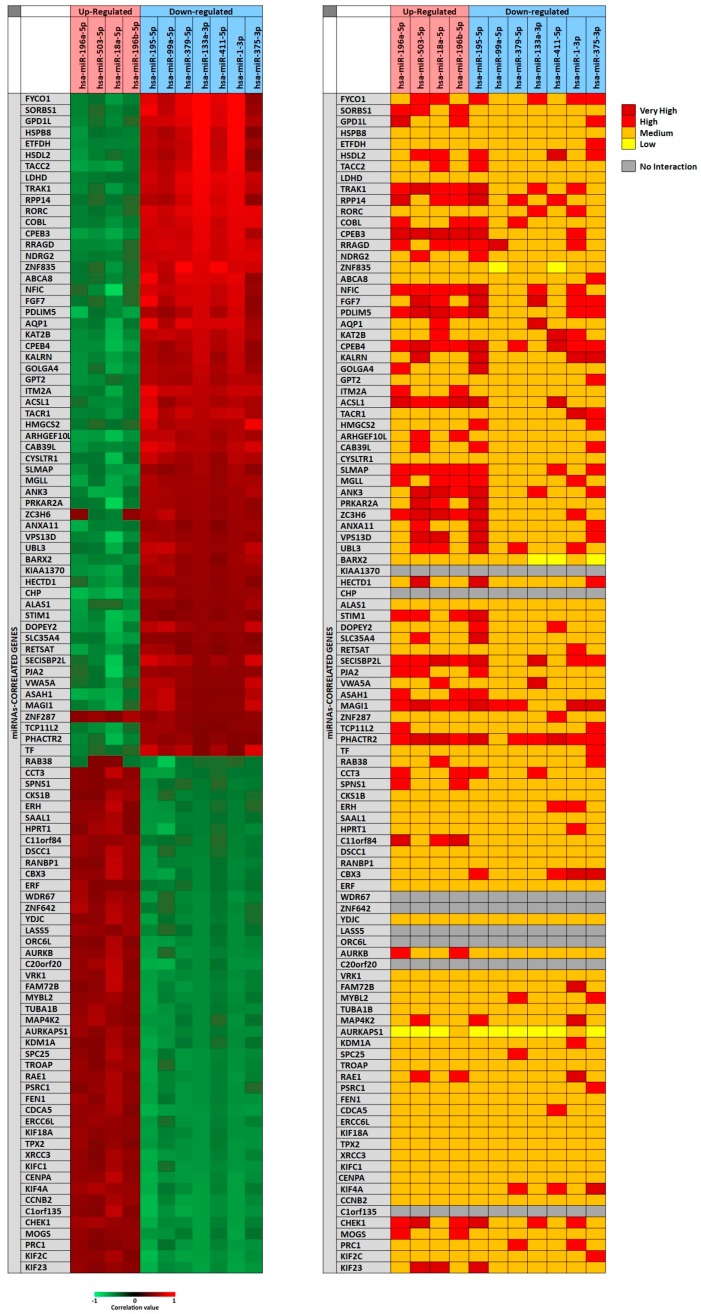
Panel (**A**) miRCancerdb analysis of genes whose expression is positively and negatively related to the 11 selected miRNAs; panel (**B**) mirDIP analysis of interaction levels between miRNAs and related genes.

In particular, for each miRNA a list of miRNAs-correlated genes, ranging from 4493 to 9042, was obtained through miRCancerdb analysis. Subsequently, these lists of genes were compared showing a total of 121 genes in common and altered by the 11 selected miRNAs. However, only the genes shared by the 11 miRNAs and belonging to the first quartile of the genes most positively and negatively correlated to each miRNA were considered (Figure 2). This selection unveiled the correlation levels of 105 different genes (Figure 2A).

In Figure 2A, the heat map showed that the down-regulated miRNAs miR-133a-3p and miR-1-3p were those with the highest positive correlation levels; instead, the miRNA with lower negative correlation levels was the up-regulated miRNA miR-18a-5p. Moreover, it can be observed that *FYCO1*, *SORBS1* and *GPD1L* genes were strongly positively correlated with the selected miRNAs; on the other hand, *ASA1*, *NFIC* and *SECISBP2L* genes were the least correlated with the analyzed miRNAs.

To further confirm the correlation levels existing among miRNAs and genes, the mirDIP tool was used. In Figure 2B, the interaction levels between the 11 selected miRNAs and the positively and negatively correlated genes are showed (Figure 2B). The figure shows that for eight genes there were no interactions with the selected miRNAs (*KIAA1370*, *CHP*, *WDR67*, *ZNF642*, *LASS5*, *ORC6L*, *C20orf20*, *C1orf135*). Overall, such analysis revealed that miR-195-5p, miR-503-5p, miR-18a-5p (up-regulated) and miR-375-3p (down-regulated) showed highest interaction levels with the 105 genes. On the other hand, the genes *CPEB3*, *CPEB4*, *MAGI1*, *PHACTR2*, *PDLIM5*, *NFIC*, *SLMAP* and *SECISBP2L* were strongly targeted by the 11 selected miRNAs (Figure 2B).

### 2.4. Determination of the Functional Roles of Tumor-Associated MiRNAs Through Pathway and GO Enrichment Analyses

For the pathway prediction analysis, all the 11 tumor-associated miRNAs were inputted into the bioinformatics prediction tool DIANA-mirPath. The analysis revealed that for the miRNAs miR-503-5p, miR-133a-3p and miR-1-3p there were not modulated pathways and targeted genes according to the TarBase Version 7.0 database of DIANA-mirPath. For the remaining miRNAs the cumulative pathway analysis showed that, overall, the miRNAs were able to alter 48 different pathways and over 2100 genes. However, the pathways involved in the tumor processes were 22 and the modulated genes amounted to 345 univocal genes (Table 4).

As shown in Table 4, the identified miRNAs play a key role in the modulation of different pathways involved in neoplastic development and in different types of tumors, highlighting their potential pro-oncogenic role when de-regulated (Table 4). The pathways found highly modulated were: “Pathways in cancer (hsa05200)”, “Cell cycle (hsa04110)”, various signal transduction pathways, including “FoxO signaling pathway (hsa04068)”, “p53 signaling pathway (hsa04115)” and “Hippo signaling pathways (hsa04390)”.

Within these pathways, *MAPK1* (18 counts), *CCND1* (17 counts), *AKT3* and *PIK3CA* (15 counts), *PIK3CB* (14 counts), *NRAS* (13 counts), *BRAF* (12 counts), *CDK4* and *CDKN1A* (11 counts) and *E2F2* (10 counts) genes were found commonly altered by the selected miRNAs. All these genes, when de-regulated, were notoriously involved in cancer development and progression.

To further confirm the functional roles of miRNAs and their modulated genes, gene enrichment analyses were performed on both miRCancerdb and DIANA-mirPath lists of genes by using both GO PANTHER and STRING software.

**Figure 3:**
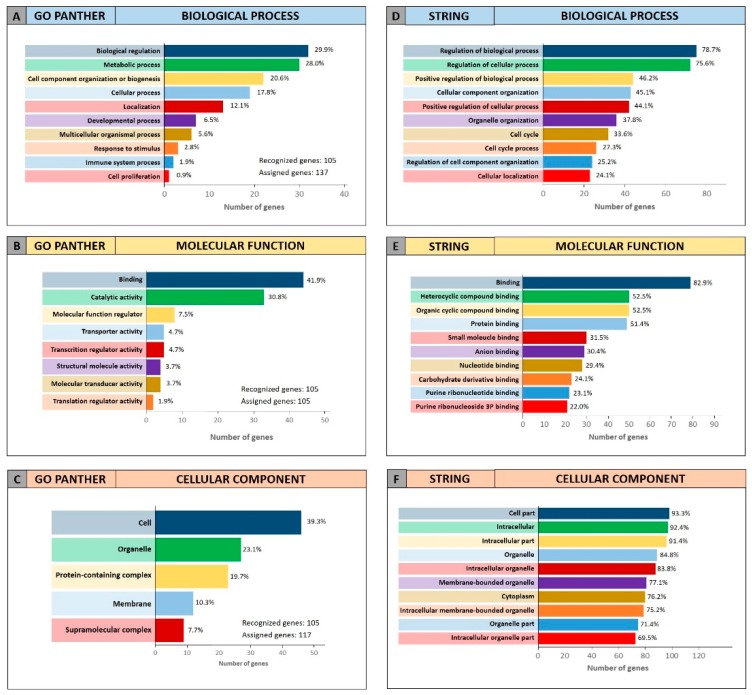
Gene Ontology enrichment of the 105 genes identified through miRCancerdb. Panel (**A**,**D**) GO PANTHER and STRING analyses of the “biological process” category; panel (**B**,**E**) GO PANTHER and STRING analyses of the “molecular function” category; panel (**C**,**F**) GO PANTHER and STRING analyses of the “cellular component” category.

Both enrichment analyses were performed on the list of the 105 miRCancerdb genes correlated to the 11 cancer-associated miRNAs giving back similar results regarding the three ontological categories “biological process”, “molecular function” and “cellular component”. Figure 3 shows the results of the GO PANTHER and STRING analyses (Figure 3).

Regarding the “biological process” category, it was demonstrated that most of the miRNAs-modulated genes are involved in the regulation of biological (29.9% and 78.7%, GO PANTHER and STRING, respectively) and cellular (17.8% and 75.6%, GO PANTHER and STRING, respectively) processes (Figure 3A,D). In Figure 3B,E, the genes were clustered according to their “molecular function” and the results showed that the genes were all involved in protein binding, cyclic compounds and nucleotides binding (STRING analysis Figure 3E). While the GO PANTHER analysis for the same category (molecular function) showed that the genes were mainly involved in the binding and, to a lesser extent, in the catalytic, molecular and transport activities (Figure 3B). Finally, with regard to the “cellular component” category, the majority of the genes were components of the cell (39.3% and 93.3, GO PANTHER and STRING, respectively) and organelles (23.1% and 84.8%, GO PANTHER and STRING, respectively; Figure 3C,F).

**Figure 4:**
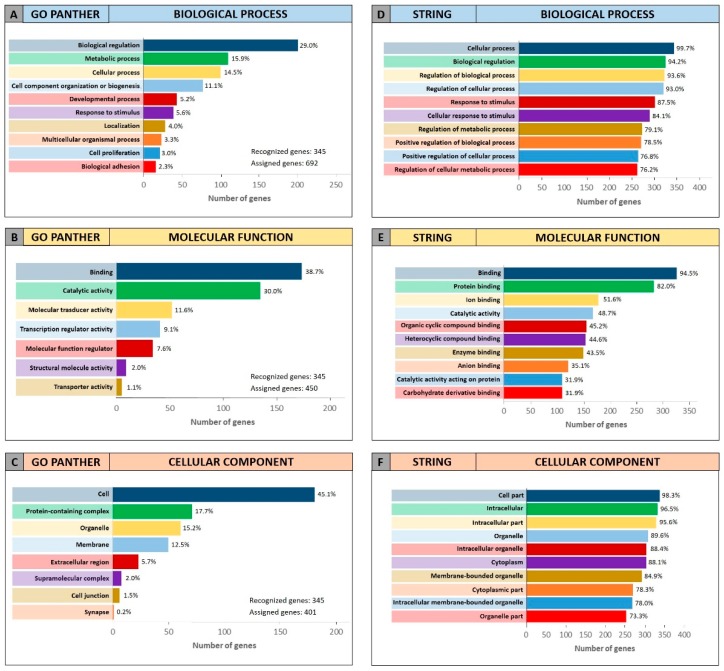
Gene Ontology enrichment of the 345 genes identified through DIANA-mirPath. Panel (**A**,**D**) GO PANTHER and STRING analyses of the “biological process” category; panel (**B**,**E**) GO PANTHER and STRING analyses of the “molecular function” category; panel (**C**,**F**) GO PANTHER and STRING analyses of the “cellular component” category.

The same GO enrichment analyses were performed on the 345 genes identified by DIANA-mirPath showing similar results to those described above (Figure 4).

Figure 4 (Panel A and D) shows that, in the “biological process” category, the genes identified by DIANA-mirPath were involved in the regulation of the biological and cellular processes as observed in Figure 3. Similarly, in the “molecular function” and “cellular component” categories, the 345 genes were involved, respectively, in molecular binding and catalytic activities (Figure 4B,E), and were components of the cell and intracellular organelles (Figure 4C,F).

### 2.5. Identification of Oral Cancer Stage-Related miRNAs

The same differential analysis performed to find the oral cancer-associated miRNAs was also performed between high-grade tumor samples (254 stage III and IV samples—high-grade) and low-grade tumor samples (94 Stage I and II samples—low-grade) in order to find oral cancer stage-related miRNAs with a prognostic significance. This second differential analysis showed that 36 miRNAs were de-regulated in high-grade samples compared to low-grade (*p* < 0.01; Table 5).

As shown in Table 5, among the 36 identified miRNAs, 31 were down-regulated and five were up-regulated. Furthermore, among the 31 down-regulated miRNAs, three were in common with those obtained by the lists of differentially expressed miRNAs in tumor samples compared to the normal one, i.e. miRNAs miR-139-3p, miR-142-5p and miR-29c-3p, which therefore may have both diagnostic and prognostic significance in oral cancers. Furthermore, the down-regulated miRNA miR-133a-3p was in common with the list of 11 miRNAs obtained from the comparison between GEO DataSets and TCGA analyses (Table 3) suggesting that these miRNAs may have both a diagnostic and prognostic role for oral cancer.

### 2.6. Prognostic Value of Oral Cancer Stage-Related miRNAs

**Figure 5:**
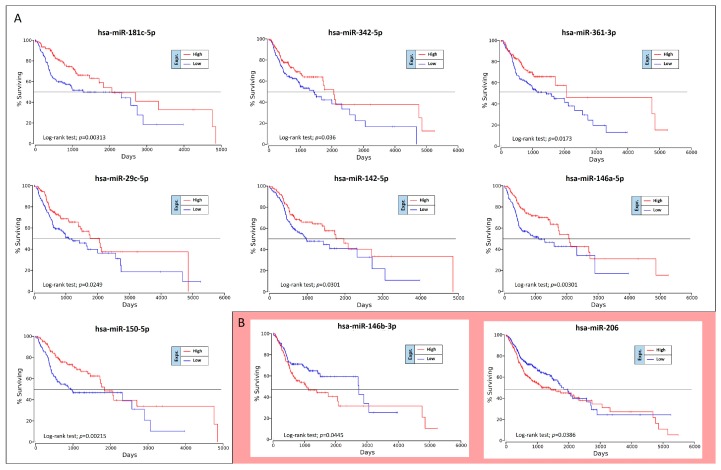
Survival analysis performed by OncoLnc. Panel (**A**) down-regulated miRNAs statistically associated with patients’ overall survival (OS) whose expression is concordant with survival curves; panel (**B**) miRNAs statistically associated with patients’ OS whose expression levels are not concordant with the survival curves.

The OncoLnc analysis performed on the 36 differently expressed miRNAs in high-grade oral cancers revealed the real prognostic significance of each miRNA in terms of patients’ overall survival (OS). As shown in Figure 5, of 36 miRNAs analyzed only nine were statistically associated with patients’ OS (log-rank test, *p* < 0.05). These prognostic miRNAs were all down-regulated miRNAs, i.e., miR-181c-5p, miR-342-5p, miR-361-3p, miR-29c-5p, miR-142-5p, miR-146a-5p, miR-150-5p, miR-146b-3p and miR-206 (Figure 5).

However, two of these miRNAs, miR-146b-3p and miR-206, have shown results of dubious interpretation. In fact, despite these miRNAs are down-regulated in high-grade tumors, their down-regulation is not associated with a worse OS, but with a better prognosis (Figure 5B).

To confirm the Kaplan-Meier results obtained by using OncoLnc, the OS curves were also calculated by using GraphPad v.6 and analyzing the TGCA HNSC survival data previously downloaded from the UCSC Xena Browser. Overall, this analysis revealed the same results previously obtained.

**Figure 6:**
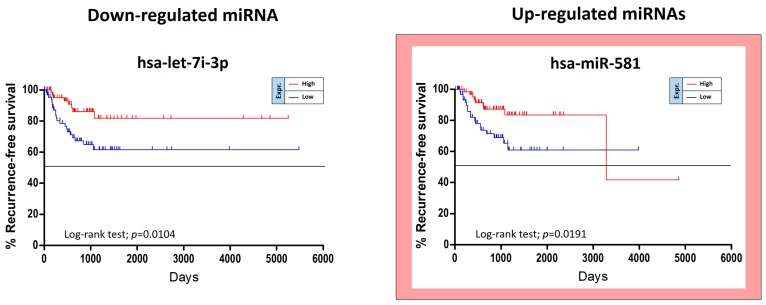
Recurrence-free survival analysis performed on the TCGA HNSC data.

The TCGA HNSC data were also used for the identification of miRNAs able to predict the risk of oral cancer recurrence. For this purpose, GraphPad Kaplan-Meier curves showed that two out of 36 tumor stage-related miRNAs were statistically linked to the patients’ recurrence-free survival (RFS). Of these miRNAs, miR-581 was up-regulated and miR-let-7i-3p was down-regulated. Unexpectedly the over-expression of the up-regulated miR-581 was not associated with a worse prognosis, but with a minor RFS (Figure 6).

Other five miRNAs, miR-151a-5p, miR-6718-5p, miR-660-5p, miR-4772-3p and miR-217-5p, showed a weak correlation with RFS when de-regulated, however no statistical significance was reached.

These analyses allowed us to identify 11 miRNAs significantly associated to both tumor grade and patients’ OS and RFS, of these only eight were related to patients’ OS (seven miRNAs) and RFS (one miRNA), respectively.

### 2.7. Determination of the Functional Roles of the 11 Tumor-Grade Associated miRNAs Through Pathway and GO Enrichment Analyses

**Figure 7:**
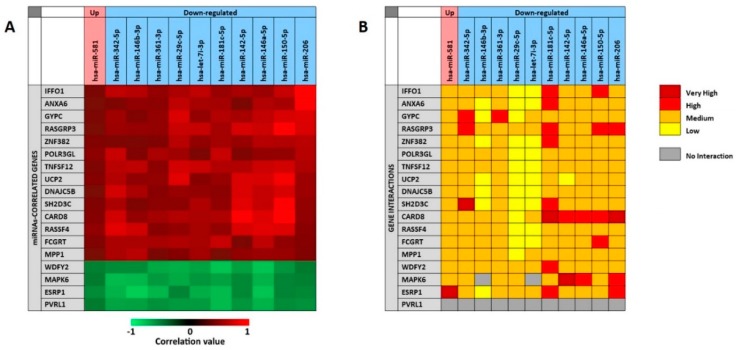
Panel (**A**) miRCancerdb analysis of genes whose expression is positively and negatively related to the 11 selected miRNAs; panel (**B**) mirDIP analysis of interaction levels between miRNAs and related genes.

As previously described for the analysis of the 11 selected miRNAs associate to the presence of tumor, the miRCancerdb and mirDIP analyses were performed for the 11 miRNAs associated to patients’ prognosis in order to identify the miRNAs-correlated and -targeted genes. The miRCancerdb analysis showed that 19 different genes were positively and negatively correlated to the 11 selected miRNAs (Figure 7A).

The heat map showed that the down-regulated miRNAs miR-150-5p and miR-206 are those with the highest positive correlation levels. Of note, these two miRNAs were also those with the higher levels of down-regulation among the 36 differentially expressed miRNAs showed in Table 5. Figure 7 also shows that the miRNAs miR-181c-5p and miR-146a-5p were those more negatively correlated with the identified genes. By considering the genes, it was observed that *CARD8* and *RASGAP3* genes were those that were more positively correlated with the selected miRNAs, while the four genes *WDFY2, MAPK6, ESRP1* and *PVRL1* were all negatively correlated with the 11 miRNAs with similar correlation levels.

The mirDIP analysis performed on the 19 genes and the 11 miRNAs showed that for the *PVRL1* gene no interaction levels were available. Overall, the analysis revealed the existence of medium interaction levels between miRNAs and genes. However, the down-regulated miR-29c-5p and let-7i-3p showed lower interaction levels with most of the 19 genes, while the miR-181c-5p was the miRNA with the higher interaction levels. On the other hand, the *CARD8* gene was the most targeted by the 11 selected miRNAs, while the *UCP2* was the less targeted (Figure 7B).

After the gene targets analysis, the DIANA-mirPath analysis of the 11 prognostic miRNAs revealed that for the miRNA miR-581 there were not modulated pathways and targeted genes according to the TarBase Version 7.0 database of the mirPath tool. For the other 10 miRNAs the cumulative pathway analysis showed that, the miRNAs were able to modulate 44 different pathways and over than 1300 genes. The selection of the 21 pathways involved in the tumor processes showed that the selected miRNAs were able to modulate 292 univocal genes (Table 6).

The DIANA-mirPath analysis showed that all the selected miRNAs were involved in the modulation of the “PI3K-Akt signaling pathway (hsa04151)” and the “Cell cycle (hsa04110)”, both involved in various neoplastic processes when altered (Table 6). Of note, “Cell cycle (hsa04110)” pathways were also strongly altered by the 11 cancer-associated miRNAs previously analyzed (Table 4). The genes altered by these miRNAs were all involved in neoplastic processes, such as *CCND1* (18 counts), *MAPK1* (16 counts), *MAP2K1* (14 counts), *PIK3CB* and *PIK3R3* (16 counts), *AKT2* and *AKT3* (15 counts), *CDK4* and *CDK6* (11 counts), etc.

The genes identified through miRCancerdb and DIANA-mirPath analyses were finally analyzed with GO PANTHER and STRING to establish for which molecular processes and functions these miRNAs were enriched.

**Figure 8:**
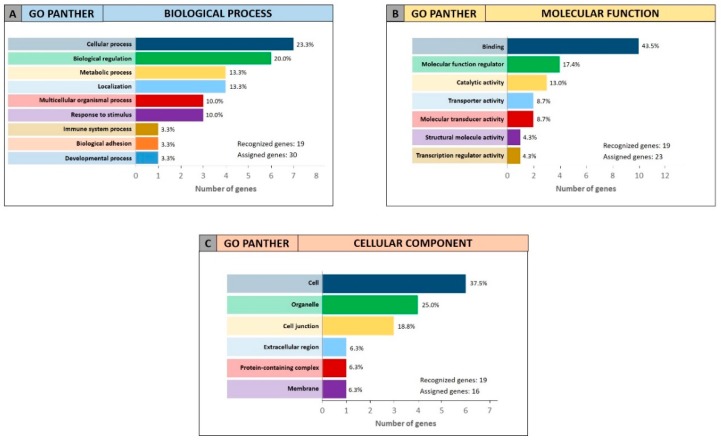
Gene Ontology enrichment of the 19 genes identified through miRCancerdb. Panel (**A**) GO PANTHER analysis of the “biological process” category; panel (**B**) GO PANTHER analysis of the “molecular function” category; panel (**C**) GO PANTHER analysis of the “cellular component” category.

For the 19 genes identified by miRCancerdb analysis only the GO PANTHER evaluation was performed because the gene ontology enrichment performed by STRING requires a wide number of analyzed genes. The GO PANTHER analysis showed that most of selected genes were involved in the cellular processes (23.3% of genes) and in biological regulation (20.0% of genes) for the “biological process” category (Figure 8A). Regarding the “molecular function” category, the analysis demonstrated that the 43.5% and 17.4% of genes were involved in binding and molecular regulatory functions, respectively (Figure 8B); while for the “cellular component” category, the results showed that the 19 genes constitute mainly part of the cell, of the organelles and of the cell junctions (37.5%, 25.0% and 18.8% respectively; Figure 8C).

The same enrichment analysis was performed on the 292 genes identified by DIANA-mirPath carrying out both GO PANTHER and STRING analyses.

**Figure 9:**
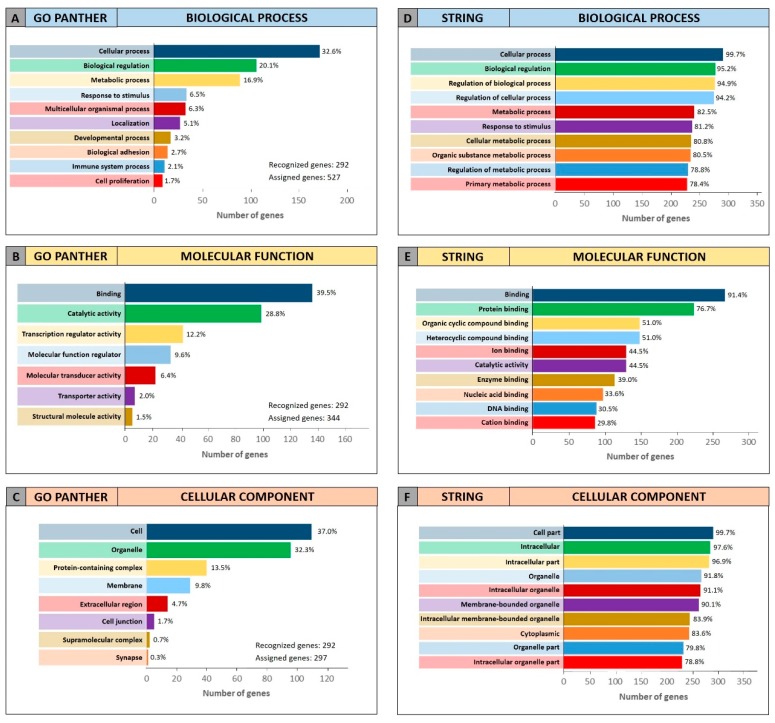
Gene Ontology enrichment of the 345 genes identified through DIANA-mirPath. Panel (**A**,**D**) GO PANTHER and STRING analyses of the “biological process” category; panel (**B**,**E**) GO PANTHER and STRING analyses of the “molecular function” category; panel (**C**,**F**) GO PANTHER and STRING analyses of the “cellular component” category.

The results obtained for the “biological process” category showed that the identified genes were mainly involved in the cellular processes (23.3% of genes) and in biological regulation (20.0% of genes) as observed for the 19 genes identified by miRCancerdb (Figure 9A,D). Furthermore, both the analyses (GO PANTHER and STRING) showed that the 292 genes were involved in molecular binding and catalytic activities (Figure 9B,E), as observed in the previous evaluations. Regarding the “cellular component” category, it was finally demonstrated that the genes were part of the cell and of the intracellular organelles (Figure 9C,F).

## 3. Discussion

During the last decade, the advancement of bioinformatics and high-throughput technologies led to the development of omics sciences, as well as to the collection of thousands of petabytes of molecular data related to various human diseases, including tumors [39].

The increase in the number of available bioinformatics data allowing the understanding of various physio-pathological aspects of tumors. However, the huge amount of data, either deriving from individual basic science experiments, or collected by large international consortia, such as TCGA and ENCODE, are often incorrectly analyzed, thus generating conflicting results [40,41,42].

In order to best analyze the so-called “Big Data”, in recent years different researchers have created several bioinformatics software useful for a fast and efficient analysis of a large number of data thus interpretation through a process named “data mining” [43,44].

Thanks to the availability of new software for the computational analysis of Big Data, numerous studies tried to establish the molecular mechanisms responsible for neoplastic transformation, as well as to identify novel molecular targets or biomarkers useful for the management of tumors [45].

In recent years, several genetic, epigenetic and proteomic data were also generated for oral cancer. These data allowed the researchers to obtain important information regarding the main molecular and clinical-pathological characteristics of this kind of tumor. However, the analysis of the data contained in the various oral cancer datasets generated conflicting data difficult to interpret due to the lack of data integration among the different data matrices [46]. Furthermore, despite the increasing number of bioinformatics studies, no effective diagnostic and prognostic biomarkers have been yet identified for oral cancers, making this pathology one of the most aggressive, since in most cases it is not promptly diagnosed [47].

Therefore, the aim of the present study was to identify new specific diagnostic and prognostic biomarkers for oral cancer through the analysis and integration of different miRNAs profiling datasets, using several computational approaches.

For this purpose, two of the biggest worldwide genomics databases, TCGA and GEO DataSets were analyzed in order to select miRNA expression profiling datasets. In particular, the analysis of the TCGA HNSC “miRNA mature strand expression RNAseq by Illumina Hiseq” dataset and of two GEO DataSets miRNA microarray matrices allowed the identification of a panel of miRNAs with diagnostic and prognostic value for oral cancer patients. From these datasets, a group of 11 de-regulated miRNAs was identified by comparing cancer patients with healthy controls. Among these miRNAs, the up-regulated miR-196a-5p and miR-196b-5p and the down-regulated miR-99a-5p, miR-133a-3p, miR-1-3p and miR-375-3p were the most de-regulated and therefore these miRNAs may be used to improve the actual diagnostic strategies for oral cancers. Indeed, different research groups are currently investigating all these miRNAs because their deregulation is associated with the development of different cancers. In particular, Sutliff and colleagues (2019) demonstrated that the de-regulation miR-196 family (miR-196a-5p and miR-196b-5p) is associated with the development of lung cancer [48].

Furthermore, other studies have demonstrated that the miR-196 family and other miRNAs, including miR-375 and miR-133a-3p, may play a key role as diagnostic biomarkers for head and neck cancers, especially for the tumors of the oral cavity [49,50,51]. In addition to these five miRNAs, the miRNAs miR-139-3p, miR-142-5p and miR-29c-3p are also noteworthy because beyond their diagnostic role, they have also an important prognostic role. On this regard, the analysis of miRNA expression levels in high-grade tumors compared to the low-grade tumors contained in the TCGA HNSC dataset revealed that the down-regulation of these three miRNAs, together with the aforementioned miRNA miR-133a-3p, was associated with a more aggressive and infiltrating phenotype. Accordingly, these results are supported by several studies performed on different tumors where it has been shown that the de-regulation of these miRNAs is associated with a more aggressive tumor phenotype [32,52,53,54].

Furthermore, the OncoLnc analysis revealed that among the 36 tumor-stage associated miRNAs only eight were related to patients’ OS and RFS (seven and one, respectively). Of note, all these miRNAs were found all down-regulated in high-grade tumors compared to low-grade. In particular, the most statistically significant miRNAs associated with OS, and therefore to a worse prognosis, were the miRNAs miR-150-5p, miR-181c-5p and miR-146a-5p. Regarding the RFS, only the miRNA miR-let-7i-3p was a good indicator of disease recurrence. These data are also supported by several studies since the miR-7i (both 3p and 5p strands) is associated with a poorer prognosis when down-regulated [55]. Furthermore, other studies showed that the down-regulation of other miRNAs, such as miR-375 and miR-181c, is associated with a cancer aggressive phenotype [56,57].

Therefore, these first computational data demonstrated that by using an integrated computational approach for the analysis of miRNAs datasets it is possible to identify a set of miRNAs potentially used as specific biomarkers for oral cancers. As described above, the validity of the obtained results is further strengthened by the results obtained by other research groups in independent experimental studies.

Once identified which miRNAs bear a diagnostic and/or prognostic significance, it was also established which genes and pathways they were able to modulate to uncover their functional roles. As described in previous studies, the DIANA-mirPath analysis showed that the computationally selected miRNAs were strictly related to cancer development since they were able to alter key oncogenic pathways [32,33,34,58]. On this regard, the oral cancer-associated miRNAs identified in the present study were able to alter several intracellular signal transduction pathways, including mTOR, p53 and TGF-β pathways, whose implication in the development of oral carcinoma has been widely demonstrated [59,60,61]. Similarly, the selected miRNAs were also able to target genes frequently down-regulated or over-expressed in oral cancers. Some of these genes, including *AKT*, *BRAF*, *PIK3CA*, *NRAS*, *GSK3*, *CNND1*, etc., are involved not only in oral cancers but, generally, in several solid tumors [62,63,64,65]. The subsequent gene ontology enrichment analyses further demonstrated that the miRNAs’ targeted genes were involved in the biological process linked to the cell proliferation, biological regulation, protein binding, catalytic activities and metabolic processes.

Similarly, the prediction and GO enrichment analyses performed on the 11 miRNAs associated to the patients’ OS and RFS revealed that all the miRNAs with a significant diagnostic and/or prognostic role were able to modulate several cancer pathways by modulating numerous genes known to be involved in neoplastic transformation.

Overall, the computational approaches adopted in the present studies allowed us to identify a set of specific miRNAs for the diagnosis of oral cancer and the definition of patients’ prognosis through the integrated analysis of different bioinformatics datasets that allowed us to understand the functional role of each miRNA. However, the results obtained from this study represent only the starting point for identifying effective markers for oral carcinoma. Therefore, further experimental and functional studies will have to be performed on a large number of samples in order to evaluate the expression levels of these putative miRNAs biomarkers and to validate their predictive role for oral cancer. With the advancement of both bioinformatics and high-throughput and high-sensitive molecular technologies this future goal can be easily achieved thanks to the detection of even small variations in the expression levels of selected miRNAs indicative of the presence of a possible pathological state [66,67,68].

## 4. Materials and Methods

### 4.1. Oral Cancer MicroRNA Datasets Selection

In order to identify miRNAs potentially involved in the development and progression of oral cancer, several oral cancer miRNAs datasets were taken into account. Firstly, the oral cancer datasets of microRNA profiling by array were selected by checking within the datasets registered in the GEO DataSets portal publicly available on NCBI (www.ncbi.nlm.nih.gov/geo/) [69]. In particular, for the selection of the suitable datasets an advanced search was carried out by inserting the search terms “((“non coding RNA profiling by array”[DataSet Type]) and oral carcinoma) and “Homo sapiens”[porgn:__txid9606]”. With this first approach, a list of all oral cancer datasets containing miRNA expression levels was obtained. Of these datasets, only those that respect the following inclusion and exclusion criteria were selected for the subsequent evaluations:

Inclusion criteria, i) datasets containing miRNA expression levels of oral cancer tissues, excluding tumor arising in the hypopharynx, larynx, esophagus and tonsil; ii) datasets reporting miRNAs expression levels of both tumor and normal tissue samples; iii) datasets containing the miRNA expression data of at least 30 samples (tumor + normal).

Exclusion criteria, i) datasets constructed only with tumor samples; ii) datasets containing information about miRNAs of oral cancer or normal cell lines; iii) datasets containing information on miRNAs expression levels of serum samples.

The search criteria for the selection of the datasets contained in the GEO DataSets database allowed us to preliminarily identify 37 different datasets of oral carcinoma microRNA profiling by array (published up to December 2018). However, most of these datasets did not respect the exclusion and inclusion criteria because they were datasets reporting the miRNA expression data relative to tumor cell lines and not from oral cancer patients. Hence, after the application of the abovementioned criteria only two datasets were selected for performing the differential analyses (Table 7).

In addition to the datasets contained in the GEO DataSets database, also the TCGA Head and Neck Cancer (HNSC) database was selected. Among the 25 datasets available in the TCGA HNSC database, the “Phenotype” and “miRNA mature strand expression RNAseq by Illumina Hiseq” HNSC datasets were downloaded for the analyses by using the UCSC Xena Browser (https://xenabrowser.net/) portal where all the HNSC molecular profiling data, generated by the TCGA consortium, were deposited, including those of oral cancer. In particular, the first dataset contained the clinical-pathological data of 604 samples (530 cancer patients and 74 normal individuals) while the second one contained the miRNAs expression profile of 529 samples. Since the TCGA HNSC database also contains tumor samples obtained not only from the oral cavity but also from other sites (oropharynx, hypopharynx, larynx and tonsil), for the purposes of this study only the data of samples derived from alveolar ridge, base of tongue, buccal mucosa, floor of mouth, hard palate, lip, oral cavity and oral tongue were analyzed. In this way, the number of analyzed samples was reduced to 399. By selecting only samples of the oral cavity also the number of samples with available miRNAs expression profile it was reduced passing from 529 to 351 samples.

### 4.2. Differential Analysis of miRNAs Expression Between Groups

Two distinct differential analyses were performed by using the datasets selected from the GEO DataSets and TCGA databases. A first differential analysis was performed to both GEO DataSets and TCGA data matrices by integrating the different GEO DataSets platform and by comparing the miRNAs expression levels of tumor samples with a normal one in order to identify new diagnostic biomarkers.

The second differential analysis was conducted only for the TCGA dataset comparing the expression levels of miRNAs of advanced tumors with that of low-grade tumors to identify miRNAs able to define the prognosis of patients.

In particular, the data matrices of each dataset selected from GEO DataSets were downloaded to identify the down-regulated or up-regulated miRNAs in oral cancer. The differential analysis between cancer and normal samples was performed by using the GEO2R tool [69]. The fold change value (FC) obtained for each miRNA was indicated as base-2 logarithm of FC (logFC) in order to normalize the data derived from different microarray platforms. Then, for each dataset only the differentially expressed miRNAs with a statistical significance *p* < 0.01 were taken into account. The lists of the de-regulated miRNAs of the two selected GEO DataSets platforms were subsequently compared in order to select only the miRNAs shared by the two datasets and with a logFC value greater than ±1.5.

In parallel, other differential analyses of miRNAs expression levels between tumor vs normal samples and between high-grade vs low-grade tumors of TCGA HNSC dataset were performed.

For the differential analyses, the samples were clustered according to the presence or absence of tumor (Tumor (348 samples) vs Normal (51 samples)) and according to the tumor stage (T3–T4 (319 samples) vs. T1–T2 (32 samples)). After patients’ stratification, the down-regulated and up-regulated miRNAs were identified by calculating the fold change value obtained through the differential analysis between the different clusters of samples. Of note, for some of the oral cancer patients the miRNA expression levels were missing (NA value). Therefore, in order to avoid the identification of non-representative miRNAs, for further analysis only the differentially expressed miRNAs with reported expression data for at least 50% of the patients and with *p*-value of *p* < 0.01 were selected.

Moreover, with reference to the differential analysis between tumor and normal samples, only the 25 most up-regulated and down-regulated miRNAs were considered to obtain more significant data; while for the differential analysis between high-grade and low-grade tumors all the differentially expressed miRNAs were considered.

Finally, the annotation of the TCGA HNSC miRNAs was performed using miRBase V.22 (http://www.mirbase.org/) by converting the miRNA IDs ‘MIMAT00’ in ‘hsa-miR-’.

### 4.3. Analysis of the Interaction Levels Between the Selected miRNAs and Oral Cancer-Altered Genes

After the identification of the most de-regulated miRNAs in tumor samples compared to normal samples, their functional roles were studied using different bioinformatics approaches. At first, using the data reported in the Catalogue of Somatic Mutations in Cancer (COSMIC) (http://cancer.sanger.ac.uk/cosmic), the most mutated and altered genes of oral cavity tumors were identified. Subsequently, for each of the COSMIC genes, the specificity of miRNA-gene interaction was highlighted by using the bioinformatics prediction software miRNA Data Integration Portal (mirDIP; http://ophid.utoronto.ca/mirDIP). In particular, this software is able to integrate the data related to 26 different databases for miRNAs (including miRBase, microrna.org and DIANA microT-CDS v5) allowing the users to centralize the data related to the miRNAs-target genes interactions obtaining more robust data. The levels of interaction between the miRNAs and the targeted gene are expressed as very high, high, medium and low according to the integrated score calculated by the mirDIP algorithm that combines the confidence scores from all available predictions data of the 26 different databases [70,71]. Furthermore, the expression levels of the 10 interacting genes identified with COSMIC were analyzed by performing the differential analysis of the gene expression data contained in the TCGA HNSC IlluminaHiSeq pancan normalized dataset.

### 4.4. Analysis of TCGA HNSC Genes Positively and Negatively Correlated with the Selected Tumor-Associated/Grade-Associated miRNAs

In addition to the COSMIC analysis, a global correlation analysis was also performed on the genes contained in the TCGA HNSC dataset whose expression is modulated, positively or negatively, by the selected tumor-associated miRNAs. In particular, for this analysis the bioinformatics tool miRCancerdb (https://mahshaaban.shinyapps.io/miRCancerdb/) was used. miRCancerdb is a free R software for the correlation analysis between gene expression and miRNAs levels with a web interface based on data contained in the TCGA and TargetScan databases [38]. In particular, through miRCancerdb, for each selected miRNA was obtained the correlation value (ρ) with different genes. The lists of genes generated for each miRNA were subsequently combined using the tool Draw Venn Diagrams of the Bioinformatics & Evolutionary Genomics (BEG) (http://bioinformatics.psb.ugent.be/webtools/Venn/) to identify the genes correlated and shared among all miRNAs.

However, since miRCancerdb uses interaction data between miRNAs and genes derived exclusively from the TargetScan database, the previously described mirDIP tool, that uses 26 different miRNA databases, was also used to establish the levels of miRNAs-genes interaction. These analyses were performed for the 11 miRNAs associated to the presence of oral cancer and for the 11 miRNAs that after OncoLnc analysis were associated to both tumor grade and patients’ OS and RFS.

### 4.5. Prediction Pathway Analysis, Gene Ontology (GO) and Functional Roles of Tumor-Associated Selected miRNAs

To better understand the functional role of the tumor-associated selected miRNAs, a pathway prediction analysis was performed. For this purpose, the bioinformatics tool DIANA-mirPath v.3 was used [72]. With this computational approach it was possible to identify the main molecular pathways altered by selected miRNAs, especially those related to tumor development and hence to oral carcinoma.

Finally, the functional role of the selected miRNAs was determined by performing the pathways enrichment analysis of the lists of genes obtained from the miRCancerdb and DIANA-mirPath v.3 analyses. For this purpose, both GO PANTHER version 14.0 (http://pantherdb.org/) and STRING version 11.0 (https://string-db.org/) software were used [73,74]. The two software were used to perform a more robust analysis. In fact, STRING database uses a number of functional classification systems including GO, Pfam and KEGG and therefore provide more comprehensive results than those obtained with the GO PANTHER analysis. Furthermore, the data derived from the biological functional prediction analyses performed with DIANA-mirPath, GO PANTHER and STRING tools are already normalized with data used as reference or negative control, therefore, no additional datasets were used for the normalization of the data.

The DIANA-mirPath, GO PANTHER and STRING analyses were performed for the 11 selected miRNAs associated to the presence of oral cancer and for the 11 miRNAs that after OncoLnc analysis were associated to both tumor grade and patients’ OS and RFS.

### 4.6. Kaplan-Meier Estimate of Overall Survival (OS) and Recurrence-Free Survival (RFS) in Patients with Down-regulated and Up-regulated Tumor Stage-Related miRNAs

In order to establish the prognostic significance of the tumor stage-related miRNAs identified, the bioinformatics tool OncoLnc (http://www.oncolnc.org/) was used [75]. OncoLnc is a tool able to derive the mortality data from the TCGA datasets, including that of HNSC, allowing the user to obtain the Kaplan-Meier survival curves for each miRNA. The software identifies which of the selected tumor stage-related miRNAs were correlated to a patients’ overall survival (OS). The OncoLnc analysis was performed according to the instruction given by the software developers that suggest to perform the analysis between the expression levels of bottom quartile samples and top quartile samples.

To further confirm the OS Kaplan-Meier results obtained by OncoLnc, the survival curves were also calculated by using the TGCA survival data downloaded only for the oral cancer (excluding tumor arising in hypopharynx, oropharynx, larynx and tonsil) analyzed with GraphPad v.6.

Furthermore, to our best knowledge no bioinformatics tools are available for the analysis of TCGA recurrence-free survival data; therefore, the RFS curves were calculated by using the TGCA HNSC progression data analyzed with a GraphPad survival curve sheet. In particular, RFS was calculated from the date of diagnosis to patient progression, or to the end of follow-up, whichever occurred first. The times of follow-up were different from patient to patient up to a maximum follow-up time of 5480 days, however, for some patients RFS data were not available.

### 4.7. Statistical Analyses

The miRNAs expression data derived from the GEO DataSets were already normalized by the GEO2R software, while the fold change values of TCGA HNSC miRNA expression levels were calculated through differential analysis. Student’s t-test was performed to select the differentially expressed miRNAs of the TCGA dataset with a statistical significance. The GEO2R software already calculated the p-values of the GEO DataSets data. For the Kaplan-Meier analyses, GraphPad survival sheet and log-rank non-parametric test were used. Data with a *p*-value of ≤0.05 and ≤0.01 were considered statistically significant.

## 5. Conclusions

In conclusion, in the present study the integrated analysis of different miRNA expression datasets and the use of several tools for the interpretation of bioinformatics data allowed us to identify a set of miRNAs that, after in vitro and in vivo validations, may be used in clinical practice for the early detection of pre-cancerous and cancerous oral lesions.

## Figures and Tables

**Table 1:** Up-regulated and down-regulated miRNAs in tumor samples compared to the healthy controls.

miRNA ID	GSE45238		GSE31277	
	Fold Change	*p*-Value *	Fold Change	*p*-Value *
**Up-regulated miRNAs**				
hsa-miR-196a-5p	8.096	9.45 × 10^−12^	8.132	1.42 × 10^−^^6^
hsa-miR-503-5p	5.010	4.83 × 10^−^^21^	2.622	4.69 × 10^−^^4^
hsa-miR-7-5p	3.505	9.41 × 10^−^^20^	2.297	5.00 × 10^−^^4^
hsa-miR-542-5p	3.348	9.21 × 10^−12^	2.700	1.10 × 10^−^^4^
hsa-miR-142-5p	3.323	3.98 × 10^−^^8^	2.633	2.12 × 10^−^^3^
hsa-miR-19a-3p	3.068	3.81 × 10^−^^7^	2.910	4.75 × 10^−^^4^
hsa-miR-18a-5p	2.646	2.34 × 10^−^^10^	1.554	2.66 × 10^−^^3^
hsa-miR-19b-3p	2.179	1.28 × 10^−^^5^	2.415	7.73 × 10^−^^4^
hsa-miR-32-5p	1.997	1.76 × 10^−^^5^	3.874	3.28 × 10^−^^5^
hsa-miR-196b-5p	1.791	2.05 × 10^−^^8^	1.874	2.00 × 10^−^^4^
hsa-miR-33b-5p	1.581	9.26 × 10^−^^4^	2.541	2.00 × 10^−^^3^
hsa-miR-34b-3p	1.558	1.95 × 10^−^^4^	2.079	1.13 × 10^−^^3^
**Down-Regulated miRNAs**				
hsa-miR-195-5p	−1.778	1.25 × 10^−^^12^	−1.620	1.71 × 10^−^^6^
hsa-miR-378a-5p	−1.799	9.47 × 10^−^^12^	−2.194	4.45 × 10^−^^3^
hsa-miR-363-3p	−1.869	1.56 × 10^−^^5^	−1.951	4.16 × 10^−^^5^
hsa-miR-100-5p	−1.883	8.04 × 10^−^^14^	−2.199	1.19 × 10^−^^4^
hsa-miR-328-5p	−2.471	1.18 × 10^−^^8^	−1.599	2.32 × 10^−^^3^
hsa-miR-99a-5p	−2.732	4.83 × 10^−^^16^	−2.441	7.82 × 10^−^^5^
hsa-miR-218-5p	−3.021	1.08 × 10^−^^10^	−1.853	1.72 × 10^−^^4^
hsa-miR-432-5p	−3.155	1.55 × 10^−^^13^	−1.718	3.14 × 10^−^^3^
hsa-miR-379-5p	−3.513	1.83 × 10^−^^11^	−2.345	9.63 × 10^−^^4^
hsa-miR-154-5p	−4.021	4.01 × 10^−^^13^	−1.826	2.00 × 10^−^^3^
hsa-miR-133a-3p	−4.202	6.37 × 10^−^^9^	−3.446	8.47 × 10^−^^3^
hsa-miR-487b-5p	−4.366	6.96 × 10^−^^15^	−1.899	9.71 × 10^−^^3^
hsa-miR-135a-5p	−4.910	1.11 × 10^−^^14^	−3.324	1.90 × 10^−^^3^
hsa-miR-411-5p	−5.574	3.25 × 10^−^^16^	−2.542	6.18 × 10^−^^3^
hsa-miR-1-3p	−9.783	3.47 × 10^−^^9^	−5.786	2.16 × 10^−^^3^
hsa-miR-375	−16.589	1.95 × 10^−^^17^	−3.198	5.12 × 10^−^^4^

* *p*-values were automatically obtained by using the GEO2R software by performing Student’s *t*-test.

**Table 2:** TCGA analysis of up-regulated and down-regulated miRNAs in the tumor compared to the normal samples.

miRNA ID	miRNA Name	FC Cancer vs Normal	*p*-Value *
**Up-regulated**			
**MIMAT0000226**	**hsa-miR-196a-5p**	**12.145**	**3.12 × 10^−19^**
**MIMAT0001080**	**hsa-miR-196b-5p**	**11.639**	**5.43 × 10^−20^**
MIMAT0000267	hsa-miR-210-3p	9.733	1.18 × 10^−9^
MIMAT0000089	hsa-miR-31-5p	7.684	8.42 × 10^−12^
MIMAT0004784	hsa-miR-455-3p	7.165	9.21 × 10^−18^
MIMAT0005923	hsa-miR-1269a	5.899	1.99 × 10^−11^
MIMAT0000102	hsa-miR-105-5p	5.510	9.64 × 10^−13^
MIMAT0004504	hsa-miR-31-3p	5.298	1.59 × 10^−9^
MIMAT0003882	hsa-miR-767-5p	5.294	5.40 × 10^−13^
MIMAT0000281	hsa-miR-224-5p	4.789	5.39 × 10^−11^
**MIMAT0002874**	**hsa-miR-503-5p**	**4.044**	**3.86 × 10^−19^**
MIMAT0002819	hsa-miR-193b-3p	3.407	8.17 × 10^−15^
MIMAT0005951	hsa-miR-1307-3p	3.395	1.14 × 10^−11^
MIMAT0000076	hsa-miR-21-5p	3.209	3.05 × 10^−10^
MIMAT0000266	hsa-miR-205-5p	3.040	1.64 × 10^−5^
MIMAT0016895	hsa-miR-2355-5p	3.023	6.22 × 10^−14^
MIMAT0004987	hsa-miR-944	3.020	7.56 × 10^−7^
MIMAT0005797	hsa-miR-1301-3p	2.902	6.39 × 10^−17^
MIMAT0000761	hsa-miR-324-5p	2.878	7.41 × 10^−12^
MIMAT0000758	hsa-miR-135b-5p	2.859	4.08 × 10^−8^
MIMAT0001341	hsa-miR-424-5p	2.856	4.57 × 10^−13^
**MIMAT0000072**	**hsa-miR-18a-5p**	**2.829**	**8.10 × 10^−10^**
MIMAT0001545	hsa-miR-450a-5p	2.828	1.20 × 10^−15^
MIMAT0000688	hsa-miR-301a-3p	2.807	5.32 × 10^−13^
MIMAT0003150	hsa-miR-455-5p	2.799	3.50 × 10^−12^
**Down-regulated**			
MIMAT0002870	hsa-miR-499a-5p	−3.296	3.76 × 10^−5^
**MIMAT0000733**	**hsa-miR-379-5p**	**−3.298**	**1.29 × 10^−10^**
MIMAT0002890	hsa-miR-299-5p	−3.504	8.97 × 10^−7^
**MIMAT0000461**	**hsa-miR-195-5p**	**−3.510**	**7.79 × 10^−14^**
MIMAT0022721	hsa-miR-1247-3p	−3.553	3.40 × 10^−7^
MIMAT0016847	hsa-miR-378c	−3.670	4.61 × 10^−8^
MIMAT0002171	hsa-miR-410-3p	−3.684	9.33 × 10^−12^
MIMAT0004603	hsa-miR-125b-2-3p	−3.694	1.52 × 10^−18^
MIMAT0004606	hsa-miR-136-3p	−3.797	1.08 × 10^−12^
MIMAT0004550	hsa-miR-30c-2-3p	−3.881	1.03 × 10^−12^
MIMAT0004552	hsa-miR-139-3p	−3.937	3.02 × 10^−14^
MIMAT0000099	hsa-miR-101-3p	−4.017	3.64 × 10^−23^
MIMAT0000087	hsa-miR-30a-5p	−4.132	6.93 × 10^−14^
**MIMAT0003329**	**hsa-miR-411-5p**	**−4.160**	**2.03 × 10^−10^**
MIMAT0000265	hsa-miR-204-5p	−4.519	1.28 × 10^−17^
MIMAT0000681	hsa-miR-29c-3p	−4.539	5.24 × 10^−17^
MIMAT0000064	hsa-let-7c-5p	−4.674	3.68 × 10^−22^
MIMAT0000462	hsa-miR-206	−5.228	4.62 × 10^−3^
MIMAT0000736	hsa-miR-381-3p	−5.293	5.06 × 10^−8^
MIMAT0000770	hsa-miR-133b	−5.580	3.66 × 10^−4^
MIMAT0000088	hsa-miR-30a-3p	−5.696	2.66 × 10^−13^
**MIMAT0000097**	**hsa-miR-99a-5p**	**−5.746**	**1.85 × 10^−27^**
**MIMAT0000427**	**hsa-miR-133a-3p**	**−7.055**	**2.93 × 10^−4^**
**MIMAT0000416**	**hsa-miR-1-3p**	**−10.663**	**8.80 × 10^−6^**
**MIMAT0000728**	**hsa-miR-375-3p**	**−18.183**	**1.3** **3 × 10^−11^**

In bold the miRNAs in common with the results of the GEO DataSets analysis; * *p*-values were calculated by Student’s *t*-test.

**Table 3:** Summary table of GEO DataSets and TCGA HNSC datasets “Cancer vs Normal” differential analyses.

miRNA Name	GEO DataSets				TCGA HNSC Datasets	
	GSE45238		GSE31277			
	FC Cancer vs Normal	*p*-Value *	FC Cancer vs Normal	*p*-Value *	FC Cancer vs Normal	*p*-Value **
**Up-regulated**						
hsa-miR-196a-5p	8.096	9.45 × 10^−12^	8.132	1.42 × 10^−6^	12.145	3.12 × 10^−19^
hsa-miR-196b-5p	1.791	2.05 × 10^−8^	1.874	2.00 × 10^−4^	11.639	5.43 × 10^−20^
hsa-miR-503-5p	5.010	4.83 × 10^−21^	2.622	4.69 × 10^−4^	4.044	3.86 × 10^−19^
hsa-miR-18a-5p	2.646	2.34 × 10^−10^	1.554	2.66 × 10^−3^	2.829	8.10 × 10^−10^
**Down-regulated**						
hsa-miR-379-5p	−3.513	1.83 × 10^−11^	−2.345	9.63 × 10^−4^	−3.298	1.29 × 10^−10^
hsa-miR-195-5p	−1.778	1.25 × 10^−12^	−1.620	1.71 × 10^−6^	−3.510	7.79 × 10^−14^
hsa-miR-411-5p	−5.574	3.25 × 10^−16^	−2.542	6.18 × 10^−3^	−4.160	2.03 × 10^−10^
hsa-miR-99a-5p	−2.732	4.83 × 10^−16^	−2.441	7.82 × 10^−5^	−5.746	1.85 × 10^−27^
hsa-miR-133a-3p	−4.202	6.37 × 10^−9^	−3.446	8.47 × 10^−3^	−7.055	2.93 × 10^−4^
hsa-miR-1-3p	−9.783	3.47 × 10^−9^	−5.786	2.16 × 10^−3^	−10.663	8.80 × 10^−6^
hsa-miR-375-3p	−16.589	1.95 × 10^−17^	−3.198	5.12 × 10^−4^	−18.183	1.33 × 10^−11^

* *p*-values were already calculated by GEO2R software; ** *p*-values were calculated by applying Student’s *t*-test.

**Table 4:** Pathways involved in neoplastic transformation and modulated by the 11 computationally selected miRNAs.

No.	KEGG Pathway	Up-Regulated miRNAs			Down-Regulated miRNAs		
		*p*-Value *	#Genes	#miRNAs	*p*-Value *	#Genes	#miRNAs
1	Bladder cancer (hsa05219)	2.25 × 10^−3^	14	3	2.78× 10^−3^	19	5
2	Cell cycle (hsa04110)	1.11 × 10^−2^	27	3	5.48× 10^−3^	43	6
3	Central carbon metabolism in cancer (hsa05230)	/	/	/	4.59 × 10^−2^	20	5
4	Chronic myeloid leukemia (hsa05220)	3.61 × 10^−4^	22	3	1.99 × 10^−2^	25	5
5	Colorectal cancer (hsa05210)	7.53 × 10^−5^	18	3	/	/	/
6	FoxO signaling pathway (hsa04068)	7.64× 10^−3^	28	3	4.50× 10^−3^	44	6
7	Glioma (hsa05214)	2.56× 10^−3^	16	3	3.70× 10^−3^	23	5
8	Hippo signaling pathway (hsa04390)	1.74 × 10^−11^	41	3	4.22 × 10^−8^	51	6
9	Melanoma (hsa05218)	1.48 × 10^−2^	15	3	/	/	/
10	mTOR signaling pathway (hsa04150)	/	/	/	1.82 × 10^−2^	22	5
11	Non-small cell lung cancer (hsa05223)	2.54 × 10^−2^	14	3	/	/	/
12	p53 signaling pathway (hsa04115)	1.84× 10^−3^	19	3	6.53 × 10^−4^	28	6
13	Pancreatic cancer (hsa05212)	2.90 × 10^−2^	17	3	4.79 × 10^−2^	23	5
14	Pathways in cancer (hsa05200)	1.33 × 10^−3^	62	3	1.68 × 10^−4^	111	6
15	Prostate cancer (hsa05215)	3.73 × 10^−2^	19	3	3.83× 10^−3^	33	6
16	Proteoglycans in cancer (hsa05205)	2.13 × 10^−4^	35	3	1.11 × 10^−12^	73	6
17	Renal cell carcinoma (hsa05211)	/	/	/	1.65 × 10^−2^	23	6
18	Small cell lung cancer (hsa05222)	2.34 × 10^−2^	19	3	1.65 × 10^−2^	29	5
19	TGF-beta signaling pathway (hsa04350)	8.01 × 10^−6^	19	3	6.45× 10^−3^	26	6
20	Thyroid cancer (hsa05216)	3.68 × 10^−2^	7	3	/	/	/
21	TNF signaling pathway (hsa04668)	/	/	/	1.88 × 10^−2^	36	6
22	Viral carcinogenesis (hsa05203)	1.53 × 10^−2^	35	3	3.77 × 10^−6^	65	6

* *p*-values were already calculated by the DIANA-mirPath by automatically applying the Fisher’s Exact Test.

**Table 5:** TCGA analysis of up-regulated and down-regulated miRNAs in high-grade compared with low-grade tumors.

miRNA ID	miRNA Name	FC High-Grade vs Low-Grade	*p*-Value **
**Up-regulated**			
MIMAT0001536	hsa-miR-429	1.279	3.20 × 10^−3^
MIMAT0003233	hsa-miR-551b-3p	1.205	1.31 × 10^−3^
MIMAT0004697	hsa-miR-151a-5p	1.172	3.78 × 10^−3^
MIMAT0003246	hsa-miR-581	1.078	3.88 × 10^−3^
MIMAT0019931	hsa-miR-4775	1.064	1.31 × 10^−3^
**Down-regulated**			
MIMAT0004594	hsa-miR-132-5p	−1.141	4.88 × 10^−3^
MIMAT0000727	hsa-miR-374a-5p	−1.148	7.65 × 10^−3^
MIMAT0022272	hsa-miR-664b-3p	−1.159	9.42 × 10^−4^
MIMAT0000415	hsa-let-7i-5p	−1.180	2.57 × 10^−3^
MIMAT0003338	hsa-miR-660-5p	−1.202	5.19 × 10^−3^
MIMAT0004775	hsa-miR-502-3p	−1.206	4.61 × 10^−3^
MIMAT0000082	hsa-miR-26a-5p	−1.209	4.96 × 10^−3^
MIMAT0004694	hsa-miR-342-5p	−1.213	4.45 × 10^−3^
MIMAT0004766	hsa-miR-146b-3p	−1.222	7.84 × 10^−3^
MIMAT0025849	hsa-miR-6718-5p	−1.223	3.61 × 10^−4^
MIMAT0004682	hsa-miR-361-3p	−1.224	8.00 × 10^−4^
MIMAT0004597	hsa-miR-140-3p	−1.232	5.38 × 10^−5^
MIMAT0004673	hsa-miR-29c-5p	−1.234	1.88 × 10^−3^
MIMAT0002808	hsa-miR-511-5p	−1.246	9.53 × 10^−3^
MIMAT0000250	hsa-miR-139-5p	−1.248	2.75 × 10^−3^
MIMAT0004585	hsa-let-7i-3p	−1.251	7.09 × 10^−3^
MIMAT0019071	hsa-miR-4532	−1.256	3.56 × 10^−3^
MIMAT0019927	hsa-miR-4772-3p	−1.258	6.01 × 10^−3^
MIMAT0000258	hsa-miR-181c-5p	−1.267	1.17 × 10^−3^
MIMAT0004570	hsa-miR-223-5p	−1.285	7.97 × 10^−3^
MIMAT0000086	hsa-miR-29a-3p	−1.290	1.94 × 10^−3^
**MIMAT0004552**	**hsa-miR-139-3p**	**–1.314**	**2.04 × 10^−3^**
**MIMAT0000433**	**hsa-miR-142-5p**	**–1.329**	**3.76 × 10^−3^**
MIMAT0000646	hsa-miR-155-5p	−1.349	5.08 × 10^−3^
MIMAT0000274	hsa-miR-217-5p	−1.354	6.83 × 10^−3^
MIMAT0000449	hsa-miR-146a-5p	−1.375	1.56 × 10^−3^
MIMAT0000280	hsa-miR-223-3p	−1.397	1.40 × 10^−3^
**MIMAT0000681**	**hsa-miR-29c-3p**	**–1.430**	**1.90 × 10^−3^**
MIMAT0000451	hsa-miR-150-5p	−1.644	3.98 × 10^−4^
* MIMAT0000427	**hsa-miR-133a-3p**	**–2.168**	**6.39 × 10^−3^**
MIMAT0000462	hsa-miR-206	−3.070	1.29 × 10^−3^

In bold, miRNAs detected in the differential analysis “Cancer vs Normal” performed in both the TCGA and GEO Datasets; * miRNA included in the list of 11 selected miRNAs; ** *p*-values were calculated by Student’s *t*-test.

**Table 6:** Tumor pathways modulated by the 11 computationally selected miRNAs associated to patients’ prognosis.

N.	KEGG Pathway	*p*-Value *	#Genes	#miRNAs
1	PI3K-Akt signaling pathway (hsa04151)	8.22 × 10^−3^	82	10
2	Cell cycle (hsa04110)	1.91 × 10^−5^	45	10
3	Proteoglycans in cancer (hsa05205)	6.81 × 10^−5^	48	9
4	Transcriptional misregulation in cancer (hsa05202)	2.00 × 10^−2^	46	9
5	FoxO signaling pathway (hsa04068)	2.66 × 10^−3^	43	9
6	Hippo signaling pathway (hsa04390)	9.14 × 10^−4^	39	9
7	Melanoma (hsa05218)	7.56 × 10^−3^	22	9
8	Viral carcinogenesis (hsa05203)	1.08 × 10^−6^	62	8
9	Prostate cancer (hsa05215)	6.33 × 10^−3^	29	8
10	Small cell lung cancer (hsa05222)	2.63 × 10^−3^	29	8
11	Renal cell carcinoma (hsa05211)	6.92 × 10^−6^	27	8
12	Chronic myeloid leukemia (hsa05220)	7.67 × 10^−4^	26	8
13	Glioma (hsa05214)	1.74 × 10^−4^	23	8
14	TGF-beta signaling pathway (hsa04350)	7.03 × 10^−4^	23	8
15	Pancreatic cancer (hsa05212)	3.18 × 10^−2^	21	8
16	Non-small cell lung cancer (hsa05223)	8.22 × 10^−3^	18	8
17	p53 signaling pathway (hsa04115)	2.00 × 10^−3^	26	7
18	Central carbon metabolism in cancer (hsa05230)	1.96 × 10^−5^	24	7
19	Colorectal cancer (hsa05210)	4.50 × 10^−2^	19	6
20	Acute myeloid leukemia (hsa05221)	3.52 × 10^−2^	17	6
21	Endometrial cancer (hsa05213)	4.46 × 10^−2^	16	6

* *p*-values were already calculated by the DIANA-mirPath by automatically applying the Fisher’s Exact Test.

**Table 7:** Features of the two selected datasets from GEO DataSets.

Series Accession	n. Normal	n. Cancer	Samples	Platform	Author Ref	Total Number
GSE45238	40	40	Fresh Frozen Tissues	GPL8179 Illumina Human v2 MicroRNA expression beadchip	Shiah SG et al, Cancer Res 2014	80
GSE31277	15	15	Fresh Frozen Tissues	GPL9770 Illumina miR arrays version 1.0	Severino P et al, BMC Cancer 2013	30

## Supplementary Materials

The following are available online at https://www.mdpi.com/2072-6694/11/5/610/s1, Table S1: List of 514 de-regulated TCGA HNSC miRNAs associated with the presence of tumor; Table S2: mirDIP number of prediction source and interaction levels between the 11 oral cancer-associated miRNAs and the genes identified by using COSMIC; Table S3: TCGA HNSC gene expression values of the 10 miRNA interacting genes.
