# Supplementary material for: Identification of Novel MicroRNAs and Their Diagnostic and Prognostic Significance in Oral Cancer
Source: Cancers (Basel). 2019 Apr 30;11(5):610. doi: 10.3390/cancers11050610 (PMC6562527; doi:10.3390/cancers11050610)
Supplement: Supplementary file 1 [file cancers-11-00610-s001.zip › Supplementary Table 2.docx]

**Table S2.** mirDIP number of prediction source and interaction levels between the 11 oral cancer-associated miRNAs and the genes identified by using COSMIC

| **Genes** | **miRNAs** | **hsa-miR-196a-5p** | | **hsa-miR-503-5p** | | **hsa-miR-18a-5p** | | **hsa-miR-196b-5p** | | **hsa-miR-195-5p** | | **hsa-miR-99a-5p** | | **hsa-miR-379-5p** | | **hsa-miR-133a-3p** | | **hsa-miR-411-5p** | | **hsa-miR-1-3p** | | **hsa-miR-375-3p** | |  |
| --- | --- | --- | --- | --- | --- | --- | --- | --- | --- | --- | --- | --- | --- | --- | --- | --- | --- | --- | --- | --- | --- | --- | --- | --- |
|  |  |  | | |  | |  | |  | |  | |  | |  | |  | |  | |  | |  | |
| **TP53 (43 %)** | Int. Score | | 0.359 | | 0.131 | | 0.062 | | 0.167 | | 0.195 | | 0.027 | | 0.397 | | 0.124 | | 0.028 | | 0.118 | | 0.203 | |
|  | N. Sources | | 6 | | 5 | | 4 | | 6 | | 7 | | 3 | | 10 | | 4 | | 3 | | 5 | | 7 | |
|  |  | |  | |  | |  | |  | |  | |  | |  | |  | |  | |  | |  | |
| **FAT1 (28 %)** | Int. Score | | 0.157 | | 0.179 | | 0.042 | | 0.154 | | 0.072 | | 0.122 | | 0.032 | | 0.147 | | 0.020 | | 0.346 | | 0.193 | |
|  | N. Sources | | 5 | | 5 | | 4 | | 4 | | 4 | | 4 | | 3 | | 5 | | 2 | | 8 | | 5 | |
|  |  | |  | |  | |  | |  | |  | |  | |  | |  | |  | |  | |  | |
| **CASP8 (23 %)** | Int. Score | | 0.274 | | 0.057 | | 0.062 | | 0.271 | | 0.038 | | 0.020 | | 0.104 | | 0.140 | | 0.137 | | 0.056 | | 0.168 | |
|  | N. Sources | | 8 | | 4 | | 4 | | 8 | | 3 | | 2 | | 6 | | 5 | | 5 | | 4 | | 7 | |
|  |  | |  | |  | |  | |  | |  | |  | |  | |  | |  | |  | |  | |
| **TERT (22 %)** | Int. Score | | 0.018 | | 0.125 | | 0.059 | | 0.024 | | 0.279 | | 0.020 | | 0.020 | | 0.156 | | 0.020 | | 0.039 | | 0.018 | |
|  | N. Sources | | 2 | | 5 | | 5 | | 2 | | 6 | | 2 | | 2 | | 7 | | 2 | | 3 | | 2 | |
|  |  | |  | |  | |  | |  | |  | |  | |  | |  | |  | |  | |  | |
| **NOTCH1 (20 %)** | Int. Score | | 0.177 | | 0.092 | | 0.146 | | 0.222 | | 0.123 | | 0.037 | | 0.043 | | 0.055 | | 0.028 | | 0.035 | | 0.151 | |
|  | N. Sources | | 5 | | 4 | | 5 | | 6 | | 4 | | 3 | | 4 | | 3 | | 3 | | 3 | | 4 | |
|  |  | |  | |  | |  | |  | |  | |  | |  | |  | |  | |  | |  | |
| **CDKN2A (16 %)** | Int. Score | | 0.144 | | 0.180 | | 0.046 | | 0.147 | | 0.430 | | 0.040 | | 0.061 | | 0.027 | | 0.020 | | 0.097 | | 0.029 | |
|  | N. Sources | | 5 | | 7 | | 4 | | 5 | | 10 | | 3 | | 4 | | 3 | | 2 | | 5 | | 3 | |
|  |  | |  | |  | |  | |  | |  | |  | |  | |  | |  | |  | |  | |
| **HRAS (10 %)** | Int. Score | | 0.017 | | 0.114 | | 0.019 | | 0.023 | | 0.054 | | 0.019 | | 0.020 | | 0.024 | | 0.020 | | 0.020 | | 0.036 | |
|  | N. Sources | | 2 | | 4 | | 2 | | 2 | | 4 | | 2 | | 2 | | 3 | | 2 | | 2 | | 3 | |
|  |  | |  | |  | |  | |  | |  | |  | |  | |  | |  | |  | |  | |
| **KMT2D (10 %)** | Int. Score | | 0.206 | | 0.430 | | 0.440 | | 0.222 | | 0.739 | | 0.156 | | 0.041 | | 0.126 | | 0.020 | | 0.120 | | 0.200 | |
|  | N. Sources | | 6 | | 11 | | 10 | | 9 | | 17 | | 5 | | 4 | | 4 | | 2 | | 3 | | 4 | |
|  |  | |  | |  | |  | |  | |  | |  | |  | |  | |  | |  | |  | |
| **FGFR3 (8 %)** | Int. Score | | 0.159 | | 0.253 | | 0.292 | | 0.140 | | 0.211 | | 0.907 | | 0.044 | | 0.092 | | 0.019 | | 0.156 | | 0.019 | |
|  | N. Sources | | 7 | | 13 | | 11 | | 6 | | 10 | | 21 | | 4 | | 5 | | 2 | | 5 | | 2 | |
|  |  | |  | |  | |  | |  | |  | |  | |  | |  | |  | |  | |  | |
| **PIK3CA (8 %)** | Int. Score | | 0.036 | | 0.032 | | 0.066 | | 0.042 | | 0.167 | | 0.011 | | 0.068 | | 0.123 | | 0.011 | | 0.057 | | 0.279 | |
|  | N. Sources | | 3 | | 2 | | 3 | | 3 | | 4 | | 1 | | 3 | | 3 | | 1 | | 3 | | 7 | |
|  |  |  | | |  | |  | |  | |  | |  | |  | |  | |  | |  | |  | |
