# Supplementary material for: Identification of Novel MicroRNAs and Their Diagnostic and Prognostic Significance in Oral Cancer
Source: Cancers (Basel). 2019 Apr 30;11(5):610. doi: 10.3390/cancers11050610 (PMC6562527; doi:10.3390/cancers11050610)
Supplement: Supplementary file 1 [file cancers-11-00610-s001.zip › Supplementary Table 3.docx]

**Table S3.** TCGA HNSC gene expression values of the 10 miRNA

interacting genes

|  | **Genes** | **FC Tumor vs Normal** | **p-value*** |  |
| --- | --- | --- | --- | --- |
|  | **TP53** | **-1.383** | **1.79E-04** |  |
|  | **FAT1** | **2.301** | **2.55E-06** |  |
|  | **CASP8** | **1.713** | **2.55E-06** |  |
|  | **TERT** | **3.842** | **2.54E-13** |  |
|  | NOTCH1 | -1.155 | 2.11E-01 |  |
|  | **CDKN2A** | **3.001** | **2.57E-05** |  |
|  | HRAS | 1.259 | 6.17E-02 |  |
|  | MLL2 | -1.080 | 3.90E-01 |  |
|  | FGFR3 | 1.374 | 2.94E-01 |  |
|  | **PIK3CA** | **1.455** | **9.99E-06** |  |

In bold the significantly de-regulated genes;*p-values were calculated by

Student’s t-test
