# Supplementary material for: Identification of Novel MicroRNAs and Their Diagnostic and Prognostic Significance in Oral Cancer
Source: Cancers (Basel). 2019 Apr 30;11(5):610. doi: 10.3390/cancers11050610 (PMC6562527; doi:10.3390/cancers11050610)
Supplement: Supplementary file 1 [file cancers-11-00610-s001.zip › Supplementary Table 1.docx]

**Table S1.** List of 514 de-regulated TCGA HNSC miRNAs associated with the presence of tumor

| **miRNA ID** | **miRNA name** | **FC Cancer vs Normal** | **p-value*** |
| --- | --- | --- | --- |
| **MIMAT0000226** | **hsa-miR-196a-5p** | **12.145** | **3.12E-19** |
| **MIMAT0001080** | **hsa-miR-196b-5p** | **11.639** | **5.43E-20** |
| **MIMAT0000267** | **hsa-miR-210-3p** | **9.733** | **1.18E-09** |
| **MIMAT0000089** | **hsa-miR-31-5p** | **7.684** | **8.42E-12** |
| **MIMAT0004784** | **hsa-miR-455-3p** | **7.165** | **9.21E-18** |
| **MIMAT0005923** | **hsa-miR-1269a** | **5.899** | **1.99E-11** |
| **MIMAT0000102** | **hsa-miR-105-5p** | **5.510** | **9.64E-13** |
| **MIMAT0004504** | **hsa-miR-31-3p** | **5.298** | **1.59E-09** |
| **MIMAT0003882** | **hsa-miR-767-5p** | **5.294** | **5.40E-13** |
| **MIMAT0000281** | **hsa-miR-224-5p** | **4.789** | **5.39E-11** |
| **MIMAT0002874** | **hsa-miR-503-5p** | **4.044** | **3.86E-19** |
| **MIMAT0002819** | **hsa-miR-193b-3p** | **3.407** | **8.17E-15** |
| **MIMAT0005951** | **hsa-miR-1307-3p** | **3.395** | **1.14E-11** |
| **MIMAT0000076** | **hsa-miR-21-5p** | **3.209** | **3.05E-10** |
| **MIMAT0000266** | **hsa-miR-205-5p** | **3.040** | **1.64E-05** |
| **MIMAT0016895** | **hsa-miR-2355-5p** | **3.023** | **6.22E-14** |
| **MIMAT0004987** | **hsa-miR-944** | **3.020** | **7.56E-07** |
| **MIMAT0005797** | **hsa-miR-1301-3p** | **2.902** | **6.39E-17** |
| **MIMAT0000761** | **hsa-miR-324-5p** | **2.878** | **7.41E-12** |
| **MIMAT0000758** | **hsa-miR-135b-5p** | **2.859** | **4.08E-08** |
| **MIMAT0001341** | **hsa-miR-424-5p** | **2.856** | **4.57E-13** |
| **MIMAT0000072** | **hsa-miR-18a-5p** | **2.829** | **8.10E-10** |
| **MIMAT0001545** | **hsa-miR-450a-5p** | **2.828** | **1.20E-15** |
| **MIMAT0000688** | **hsa-miR-301a-3p** | **2.807** | **5.32E-13** |
| **MIMAT0003150** | **hsa-miR-455-5p** | **2.799** | **3.50E-12** |
| MIMAT0000093 | hsa-miR-93-5p | 2.792 | 3.47E-08 |
| MIMAT0004494 | hsa-miR-21-3p | 2.787 | 8.08E-07 |
| MIMAT0016888 | hsa-miR-4326 | 2.781 | 4.82E-12 |
| MIMAT0004980 | hsa-miR-937-3p | 2.757 | 3.29E-24 |
| MIMAT0019716 | hsa-miR-4652-5p | 2.722 | 8.81E-11 |
| MIMAT0004680 | hsa-miR-130b-5p | 2.701 | 1.81E-14 |
| MIMAT0000261 | hsa-miR-183-5p | 2.685 | 8.21E-15 |
| MIMAT0000070 | hsa-miR-17-5p | 2.662 | 8.05E-08 |
| MIMAT0017950 | hsa-miR-2355-3p | 2.646 | 1.04E-11 |
| MIMAT0004509 | hsa-miR-93-3p | 2.595 | 4.38E-17 |
| MIMAT0003880 | hsa-miR-671-5p | 2.582 | 8.52E-16 |
| MIMAT0004926 | hsa-miR-708-5p | 2.578 | 1.04E-06 |
| MIMAT0005883 | hsa-miR-1293 | 2.556 | 3.93E-08 |
| MIMAT0022696 | hsa-miR-301a-5p | 2.551 | 9.18E-18 |
| MIMAT0019814 | hsa-miR-203b-3p | 2.487 | 1.78E-04 |
| MIMAT0004496 | hsa-miR-23a-5p | 2.441 | 3.75E-10 |
| MIMAT0004749 | hsa-miR-424-3p | 2.440 | 1.72E-20 |
| MIMAT0004497 | hsa-miR-24-2-5p | 2.421 | 4.61E-07 |
| MIMAT0003283 | hsa-miR-615-3p | 2.396 | 6.03E-08 |
| MIMAT0000691 | hsa-miR-130b-3p | 2.396 | 7.34E-21 |
| MIMAT0004697 | hsa-miR-151a-5p | 2.396 | 1.96E-15 |
| MIMAT0004909 | hsa-miR-450b-5p | 2.382 | 2.02E-11 |
| MIMAT0004927 | hsa-miR-708-3p | 2.352 | 1.88E-06 |
| MIMAT0000262 | hsa-miR-187-3p | 2.351 | 6.80E-07 |
| MIMAT0003885 | hsa-miR-454-3p | 2.346 | 1.21E-10 |
| MIMAT0000073 | hsa-miR-19a-3p | 2.332 | 1.76E-06 |
| MIMAT0000279 | hsa-miR-222-3p | 2.299 | 2.84E-11 |
| MIMAT0019880 | hsa-miR-4746-5p | 2.279 | 8.71E-17 |
| MIMAT0000095 | hsa-miR-96-5p | 2.269 | 3.96E-12 |
| MIMAT0001340 | hsa-miR-423-3p | 2.252 | 2.08E-09 |
| MIMAT0009199 | hsa-miR-365a-5p | 2.251 | 1.87E-28 |
| MIMAT0004514 | hsa-miR-29b-1-5p | 2.208 | 1.01E-08 |
| MIMAT0004767 | hsa-miR-193b-5p | 2.201 | 2.61E-14 |
| MIMAT0004949 | hsa-miR-877-5p | 2.190 | 7.24E-28 |
| MIMAT0009197 | hsa-miR-205-3p | 2.148 | 6.33E-07 |
| MIMAT0004946 | hsa-miR-744-3p | 2.144 | 6.64E-09 |
| MIMAT0000075 | hsa-miR-20a-5p | 2.125 | 4.90E-05 |
| MIMAT0004672 | hsa-miR-106b-3p | 2.122 | 1.26E-13 |
| MIMAT0000259 | hsa-miR-182-5p | 2.114 | 1.15E-09 |
| MIMAT0003298 | hsa-miR-629-3p | 2.109 | 2.52E-13 |
| MIMAT0001636 | hsa-miR-452-3p | 2.093 | 6.90E-10 |
| MIMAT0000252 | hsa-miR-7-5p | 2.065 | 2.35E-13 |
| MIMAT0004586 | hsa-miR-15b-3p | 2.060 | 4.01E-11 |
| MIMAT0002891 | hsa-miR-18a-3p | 2.050 | 4.52E-17 |
| MIMAT0000257 | hsa-miR-181b-5p | 2.047 | 7.26E-14 |
| MIMAT0000764 | hsa-miR-339-5p | 2.034 | 1.83E-05 |
| MIMAT0022726 | hsa-miR-1306-5p | 2.024 | 1.97E-12 |
| MIMAT0004553 | hsa-miR-7-1-3p | 2.022 | 6.46E-10 |
| MIMAT0000434 | hsa-miR-142-3p | 2.011 | 2.27E-04 |
| MIMAT0004493 | hsa-miR-20a-3p | 2.010 | 4.61E-07 |
| MIMAT0004657 | hsa-miR-200c-5p | 1.993 | 3.86E-05 |
| MIMAT0002174 | hsa-miR-484 | 1.984 | 2.29E-08 |
| MIMAT0022692 | hsa-miR-181b-3p | 1.974 | 2.20E-07 |
| MIMAT0004507 | hsa-miR-92a-1-5p | 1.961 | 5.47E-08 |
| MIMAT0004693 | hsa-miR-330-5p | 1.961 | 2.54E-09 |
| MIMAT0003218 | hsa-miR-92b-3p | 1.959 | 4.29E-05 |
| MIMAT0000092 | hsa-miR-92a-3p | 1.958 | 2.34E-08 |
| MIMAT0004518 | hsa-miR-16-2-3p | 1.941 | 7.90E-11 |
| MIMAT0004776 | hsa-miR-505-5p | 1.939 | 7.85E-08 |
| MIMAT0005825 | hsa-miR-1180-3p | 1.930 | 7.89E-08 |
| MIMAT0003257 | hsa-miR-550a-3p | 1.905 | 2.28E-17 |
| MIMAT0005895 | hsa-miR-548f-3p | 1.904 | 7.25E-26 |
| MIMAT0018101 | hsa-miR-3677-3p | 1.896 | 4.77E-10 |
| MIMAT0003393 | hsa-miR-425-5p | 1.892 | 5.30E-05 |
| MIMAT0000760 | hsa-miR-331-3p | 1.886 | 1.75E-10 |
| MIMAT0000441 | hsa-miR-9-5p | 1.885 | 6.13E-04 |
| MIMAT0022925 | hsa-miR-503-3p | 1.879 | 1.34E-22 |
| MIMAT0000091 | hsa-miR-33a-5p | 1.867 | 2.45E-03 |
| MIMAT0000753 | hsa-miR-342-3p | 1.857 | 7.44E-05 |
| MIMAT0003888 | hsa-miR-766-3p | 1.856 | 2.52E-14 |
| MIMAT0017992 | hsa-miR-3614-5p | 1.856 | 1.12E-07 |
| MIMAT0000227 | hsa-miR-197-3p | 1.853 | 4.66E-09 |
| MIMAT0002876 | hsa-miR-505-3p | 1.846 | 5.66E-10 |
| MIMAT0005792 | hsa-miR-320b | 1.832 | 8.47E-09 |
| MIMAT0003241 | hsa-miR-576-5p | 1.823 | 3.33E-09 |
| MIMAT0000762 | hsa-miR-324-3p | 1.822 | 2.23E-10 |
| MIMAT0002809 | hsa-miR-146b-5p | 1.820 | 1.11E-03 |
| MIMAT0004678 | hsa-miR-99b-3p | 1.801 | 6.51E-17 |
| MIMAT0001620 | hsa-miR-200a-5p | 1.796 | 3.22E-04 |
| MIMAT0019208 | hsa-miR-3074-5p | 1.779 | 2.81E-10 |
| MIMAT0003249 | hsa-miR-584-5p | 1.777 | 1.37E-07 |
| MIMAT0004983 | hsa-miR-940 | 1.775 | 1.36E-07 |
| MIMAT0004800 | hsa-miR-550a-5p | 1.773 | 7.08E-11 |
| MIMAT0018349 | hsa-miR-3934-5p | 1.769 | 3.82E-17 |
| MIMAT0022720 | hsa-miR-1304-3p | 1.766 | 1.97E-10 |
| MIMAT0004799 | hsa-miR-589-5p | 1.763 | 3.85E-19 |
| MIMAT0003256 | hsa-miR-589-3p | 1.761 | 1.82E-11 |
| MIMAT0000256 | hsa-miR-181a-5p | 1.757 | 2.35E-09 |
| MIMAT0003260 | hsa-miR-592 | 1.744 | 4.62E-11 |
| MIMAT0007884 | hsa-miR-1910-5p | 1.735 | 3.26E-07 |
| MIMAT0023712 | hsa-miR-6087 | 1.731 | 6.86E-04 |
| MIMAT0004484 | hsa-let-7d-3p | 1.713 | 2.22E-08 |
| MIMAT0004774 | hsa-miR-501-3p | 1.710 | 1.20E-05 |
| MIMAT0015050 | hsa-miR-323b-3p | 1.698 | 6.30E-08 |
| MIMAT0003389 | hsa-miR-542-3p | 1.688 | 1.63E-05 |
| MIMAT0000772 | hsa-miR-345-5p | 1.676 | 4.23E-06 |
| MIMAT0009196 | hsa-miR-103a-2-5p | 1.676 | 4.88E-07 |
| MIMAT0000417 | hsa-miR-15b-5p | 1.675 | 1.58E-08 |
| MIMAT0005893 | hsa-miR-1305 | 1.674 | 6.66E-14 |
| MIMAT0005452 | hsa-miR-519a-5p | 1.669 | 1.50E-09 |
| MIMAT0000510 | hsa-miR-320a-3p | 1.669 | 2.26E-06 |
| MIMAT0000068 | hsa-miR-15a-5p | 1.668 | 1.70E-04 |
| MIMAT0004780 | hsa-miR-532-3p | 1.667 | 1.03E-06 |
| MIMAT0000425 | hsa-miR-130a-3p | 1.657 | 1.47E-07 |
| MIMAT0030020 | hsa-miR-7705 | 1.656 | 3.74E-16 |
| MIMAT0022975 | hsa-miR-3934-3p | 1.653 | 2.33E-10 |
| MIMAT0025450 | hsa-miR-6499-5p | 1.648 | 2.61E-05 |
| MIMAT0025451 | hsa-miR-6499-3p | 1.646 | 6.77E-04 |
| MIMAT0018083 | hsa-miR-3662 | 1.644 | 2.54E-07 |
| MIMAT0000432 | hsa-miR-141-3p | 1.635 | 3.49E-03 |
| MIMAT0004700 | hsa-miR-331-5p | 1.631 | 2.09E-12 |
| MIMAT0004489 | hsa-miR-16-1-3p | 1.621 | 3.31E-05 |
| MIMAT0000455 | hsa-miR-185-5p | 1.609 | 5.33E-07 |
| MIMAT0003339 | hsa-miR-421 | 1.606 | 1.97E-11 |
| MIMAT0004495 | hsa-miR-22-5p | 1.596 | 5.59E-04 |
| MIMAT0004694 | hsa-miR-342-5p | 1.596 | 5.58E-07 |
| MIMAT0017994 | hsa-miR-3615 | 1.585 | 4.64E-07 |
| MIMAT0003258 | hsa-miR-590-5p | 1.559 | 1.25E-05 |
| MIMAT0026479 | hsa-miR-152-5p | 1.559 | 6.10E-11 |
| MIMAT0030017 | hsa-miR-7702 | 1.557 | 7.07E-09 |
| MIMAT0018197 | hsa-miR-3922-3p | 1.554 | 5.93E-12 |
| MIMAT0004593 | hsa-miR-130a-5p | 1.542 | 8.02E-16 |
| MIMAT0030021 | hsa-miR-7706 | 1.541 | 5.66E-08 |
| MIMAT0000617 | hsa-miR-200c-3p | 1.540 | 5.72E-03 |
| MIMAT0004611 | hsa-miR-185-3p | 1.536 | 1.81E-07 |
| MIMAT0009451 | hsa-miR-1976 | 1.533 | 2.49E-07 |
| MIMAT0004770 | hsa-miR-516a-5p | 1.532 | 3.73E-06 |
| MIMAT0004698 | hsa-miR-135b-3p | 1.529 | 1.39E-06 |
| MIMAT0004491 | hsa-miR-19b-1-5p | 1.519 | 6.36E-05 |
| MIMAT0004486 | hsa-let-7f-1-3p | 1.518 | 2.70E-09 |
| MIMAT0004558 | hsa-miR-181a-2-3p | 1.511 | 2.40E-07 |
| MIMAT0000270 | hsa-miR-181a-3p | 1.508 | 2.95E-05 |
| MIMAT0000081 | hsa-miR-25-3p | 1.503 | 1.33E-06 |
| MIMAT0002872 | hsa-miR-501-5p | 1.497 | 2.55E-05 |
| MIMAT0004588 | hsa-miR-27b-5p | 1.497 | 5.32E-05 |
| MIMAT0001635 | hsa-miR-452-5p | 1.495 | 1.13E-03 |
| MIMAT0004985 | hsa-miR-942-5p | 1.494 | 4.66E-06 |
| MIMAT0003284 | hsa-miR-616-5p | 1.487 | 1.74E-07 |
| MIMAT0000440 | hsa-miR-191-5p | 1.485 | 4.74E-06 |
| MIMAT0004699 | hsa-miR-148b-5p | 1.483 | 3.81E-09 |
| MIMAT0018115 | hsa-miR-3687 | 1.483 | 2.78E-08 |
| MIMAT0005905 | hsa-miR-1254 | 1.479 | 6.76E-10 |
| MIMAT0003312 | hsa-miR-642a-5p | 1.468 | 1.19E-05 |
| MIMAT0019746 | hsa-miR-4668-3p | 1.466 | 2.33E-06 |
| MIMAT0004560 | hsa-miR-183-3p | 1.462 | 1.20E-10 |
| MIMAT0022727 | hsa-miR-1307-5p | 1.461 | 4.07E-03 |
| MIMAT0002173 | hsa-miR-483-3p | 1.459 | 2.99E-04 |
| MIMAT0025851 | hsa-miR-6720-3p | 1.459 | 1.94E-04 |
| MIMAT0019738 | hsa-miR-4664-3p | 1.455 | 1.16E-07 |
| MIMAT0004584 | hsa-let-7g-3p | 1.454 | 1.81E-03 |
| MIMAT0005950 | hsa-miR-1306-3p | 1.453 | 7.51E-10 |
| MIMAT0018068 | hsa-miR-3648 | 1.449 | 8.47E-03 |
| MIMAT0000101 | hsa-miR-103a-3p | 1.445 | 3.95E-05 |
| MIMAT0000429 | hsa-miR-137-3p | 1.443 | 8.24E-05 |
| MIMAT0000278 | hsa-miR-221-3p | 1.443 | 3.20E-04 |
| MIMAT0000755 | hsa-miR-323a-3p | 1.439 | 5.31E-05 |
| MIMAT0004958 | hsa-miR-301b-3p | 1.439 | 1.72E-09 |
| MIMAT0004982 | hsa-miR-939-5p | 1.437 | 1.20E-13 |
| MIMAT0004810 | hsa-miR-629-5p | 1.436 | 7.65E-05 |
| MIMAT0004773 | hsa-miR-500a-5p | 1.435 | 6.40E-04 |
| MIMAT0016925 | hsa-miR-500b-5p | 1.435 | 6.57E-04 |
| MIMAT0022833 | hsa-miR-365b-5p | 1.429 | 2.09E-10 |
| MIMAT0003242 | hsa-miR-577 | 1.429 | 6.85E-03 |
| MIMAT0004599 | hsa-miR-143-5p | 1.425 | 4.84E-03 |
| MIMAT0002823 | hsa-miR-512-3p | 1.425 | 2.35E-06 |
| MIMAT0005933 | hsa-miR-1277-3p | 1.423 | 1.65E-08 |
| MIMAT0027682 | hsa-miR-6891-5p | 1.423 | 2.84E-07 |
| MIMAT0026477 | hsa-miR-128-1-5p | 1.422 | 2.48E-05 |
| MIMAT0000689 | hsa-miR-99b-5p | 1.421 | 6.70E-07 |
| MIMAT0022834 | hsa-miR-365b-3p | 1.413 | 4.07E-03 |
| MIMAT0000710 | hsa-miR-365a-3p | 1.413 | 4.10E-03 |
| MIMAT0004945 | hsa-miR-744-5p | 1.412 | 1.57E-05 |
| MIMAT0026475 | hsa-miR-210-5p | 1.411 | 1.60E-06 |
| MIMAT0019761 | hsa-miR-4677-3p | 1.400 | 5.82E-08 |
| MIMAT0018968 | hsa-miR-4449 | 1.398 | 1.23E-09 |
| MIMAT0018205 | hsa-miR-3928-3p | 1.398 | 6.43E-06 |
| MIMAT0003887 | hsa-miR-769-3p | 1.397 | 3.01E-08 |
| MIMAT0018071 | hsa-miR-3651 | 1.397 | 2.42E-03 |
| MIMAT0018993 | hsa-miR-4466 | 1.395 | 6.02E-28 |
| MIMAT0019927 | hsa-miR-4772-3p | 1.391 | 3.39E-03 |
| MIMAT0004748 | hsa-miR-423-5p | 1.388 | 9.00E-05 |
| MIMAT0027608 | hsa-miR-6854-5p | 1.384 | 3.08E-03 |
| MIMAT0009198 | hsa-miR-224-3p | 1.384 | 3.24E-05 |
| MIMAT0018110 | hsa-miR-3682-3p | 1.384 | 8.34E-08 |
| MIMAT0005901 | hsa-miR-1249-3p | 1.384 | 3.96E-05 |
| MIMAT0018356 | hsa-miR-3940-3p | 1.382 | 3.90E-08 |
| MIMAT0002808 | hsa-miR-511-5p | 1.381 | 4.31E-04 |
| MIMAT0000078 | hsa-miR-23a-3p | 1.375 | 8.23E-07 |
| MIMAT0017982 | hsa-miR-3605-3p | 1.371 | 8.31E-11 |
| MIMAT0027587 | hsa-miR-6842-3p | 1.367 | 2.59E-06 |
| MIMAT0004658 | hsa-miR-155-3p | 1.365 | 1.72E-07 |
| MIMAT0004567 | hsa-miR-219a-1-3p | 1.361 | 6.75E-04 |
| MIMAT0000069 | hsa-miR-16-5p | 1.361 | 6.36E-05 |
| MIMAT0031177 | hsa-miR-7974 | 1.356 | 4.01E-04 |
| MIMAT0000084 | hsa-miR-27a-3p | 1.356 | 2.02E-04 |
| MIMAT0018191 | hsa-miR-3917 | 1.353 | 7.62E-05 |
| MIMAT0004911 | hsa-miR-874-3p | 1.352 | 3.58E-03 |
| MIMAT0026734 | hsa-miR-942-3p | 1.351 | 2.24E-08 |
| MIMAT0017352 | hsa-miR-2277-5p | 1.345 | 4.29E-10 |
| MIMAT0003884 | hsa-miR-454-5p | 1.340 | 6.52E-09 |
| MIMAT0004498 | hsa-miR-25-5p | 1.339 | 1.68E-13 |
| MIMAT0000065 | hsa-let-7d-5p | 1.337 | 3.09E-06 |
| MIMAT0001618 | hsa-miR-191-3p | 1.336 | 1.84E-05 |
| MIMAT0003340 | hsa-miR-542-5p | 1.335 | 3.17E-05 |
| MIMAT0004614 | hsa-miR-193a-5p | 1.329 | 3.49E-04 |
| MIMAT0021021 | hsa-miR-5001-5p | 1.327 | 1.32E-09 |
| MIMAT0019820 | hsa-miR-4713-5p | 1.325 | 3.56E-07 |
| MIMAT0019696 | hsa-miR-4638-3p | 1.322 | 9.16E-08 |
| MIMAT0015045 | hsa-miR-3170 | 1.318 | 3.56E-08 |
| MIMAT0000759 | hsa-miR-148b-3p | 1.317 | 3.71E-05 |
| MIMAT0017990 | hsa-miR-3613-5p | 1.316 | 6.12E-03 |
| MIMAT0019926 | hsa-miR-4772-5p | 1.316 | 1.08E-03 |
| MIMAT0002847 | hsa-miR-518c-5p | 1.315 | 6.37E-04 |
| MIMAT0004761 | hsa-miR-483-5p | 1.310 | 4.99E-03 |
| MIMAT0026738 | hsa-miR-1287-3p | 1.309 | 2.67E-03 |
| MIMAT0019229 | hsa-miR-3940-5p | 1.308 | 1.05E-03 |
| MIMAT0005943 | hsa-miR-1292-5p | 1.306 | 5.37E-07 |
| MIMAT0014990 | hsa-miR-3127-5p | 1.305 | 6.28E-03 |
| MIMAT0003322 | hsa-miR-652-3p | 1.305 | 2.75E-04 |
| MIMAT0019729 | hsa-miR-4661-5p | 1.304 | 1.98E-04 |
| MIMAT0004503 | hsa-miR-29a-5p | 1.304 | 2.14E-03 |
| MIMAT0019776 | hsa-miR-1343-3p | 1.303 | 8.78E-11 |
| MIMAT0019725 | hsa-miR-4658 | 1.300 | 1.66E-03 |
| MIMAT0010195 | hsa-let-7a-2-3p | 1.297 | 5.52E-03 |
| MIMAT0019221 | hsa-miR-3677-5p | 1.296 | 7.78E-05 |
| MIMAT0000456 | hsa-miR-186-5p | 1.295 | 4.07E-05 |
| MIMAT0000680 | hsa-miR-106b-5p | 1.292 | 1.42E-03 |
| MIMAT0019958 | hsa-miR-4788 | 1.288 | 1.69E-05 |
| MIMAT0005796 | hsa-miR-1271-5p | 1.287 | 1.25E-03 |
| MIMAT0022708 | hsa-miR-584-3p | 1.286 | 1.11E-06 |
| MIMAT0000457 | hsa-miR-188-5p | 1.282 | 1.06E-03 |
| MIMAT0003326 | hsa-miR-663a | 1.277 | 5.04E-06 |
| MIMAT0004671 | hsa-miR-194-3p | 1.274 | 7.22E-05 |
| MIMAT0004957 | hsa-miR-760 | 1.264 | 1.10E-06 |
| MIMAT0018120 | hsa-miR-3691-5p | 1.263 | 1.65E-08 |
| MIMAT0004502 | hsa-miR-28-3p | 1.262 | 2.92E-04 |
| MIMAT0018119 | hsa-miR-3690 | 1.259 | 4.05E-03 |
| MIMAT0002821 | hsa-miR-181d-5p | 1.258 | 2.24E-04 |
| MIMAT0030429 | hsa-miR-7854-3p | 1.253 | 4.52E-05 |
| MIMAT0014979 | hsa-miR-3117-3p | 1.253 | 1.10E-04 |
| MIMAT0005882 | hsa-miR-548k | 1.251 | 1.27E-04 |
| MIMAT0004682 | hsa-miR-361-3p | 1.248 | 1.25E-03 |
| MIMAT0015053 | hsa-miR-3176 | 1.248 | 4.38E-18 |
| MIMAT0022270 | hsa-miR-5579-3p | 1.247 | 1.14E-03 |
| MIMAT0002844 | hsa-miR-518b | 1.246 | 5.61E-04 |
| MIMAT0004679 | hsa-miR-296-3p | 1.245 | 2.15E-04 |
| MIMAT0002830 | hsa-miR-520f-3p | 1.244 | 1.32E-03 |
| MIMAT0021044 | hsa-miR-5010-3p | 1.241 | 1.09E-06 |
| MIMAT0017993 | hsa-miR-3614-3p | 1.238 | 2.80E-03 |
| MIMAT0005577 | hsa-miR-1226-3p | 1.232 | 2.27E-06 |
| MIMAT0004819 | hsa-miR-671-3p | 1.222 | 2.99E-03 |
| MIMAT0018107 | hsa-miR-3680-3p | 1.221 | 3.35E-06 |
| MIMAT0005584 | hsa-miR-1229-3p | 1.215 | 1.11E-05 |
| MIMAT0000222 | hsa-miR-192-5p | 1.215 | 2.44E-03 |
| MIMAT0026718 | hsa-miR-874-5p | 1.215 | 1.19E-06 |
| MIMAT0004928 | hsa-miR-147b-3p | 1.214 | 2.35E-03 |
| MIMAT0004505 | hsa-miR-32-3p | 1.211 | 5.40E-04 |
| MIMAT0019841 | hsa-miR-4724-5p | 1.210 | 3.38E-03 |
| MIMAT0015069 | hsa-miR-3187-3p | 1.210 | 5.54E-08 |
| MIMAT0004805 | hsa-miR-616-3p | 1.204 | 1.09E-04 |
| MIMAT0003287 | hsa-miR-618 | 1.202 | 4.05E-04 |
| MIMAT0004956 | hsa-miR-374b-3p | 1.202 | 1.99E-03 |
| MIMAT0019918 | hsa-miR-4766-3p | 1.197 | 6.48E-03 |
| MIMAT0027357 | hsa-miR-6728-5p | 1.195 | 4.87E-03 |
| MIMAT0019214 | hsa-miR-3173-5p | 1.194 | 2.26E-05 |
| MIMAT0018187 | hsa-miR-3913-5p | 1.193 | 5.52E-03 |
| MIMAT0030425 | hsa-miR-7850-5p | 1.193 | 1.47E-05 |
| MIMAT0002858 | hsa-miR-520g-3p | 1.191 | 2.76E-03 |
| MIMAT0019202 | hsa-miR-3129-3p | 1.191 | 8.04E-04 |
| MIMAT0005583 | hsa-miR-1228-3p | 1.186 | 3.37E-03 |
| MIMAT0022710 | hsa-miR-659-5p | 1.185 | 3.89E-03 |
| MIMAT0022496 | hsa-miR-5703 | 1.184 | 1.11E-05 |
| MIMAT0028122 | hsa-miR-7112-3p | 1.184 | 3.54E-04 |
| MIMAT0027486 | hsa-miR-6793-5p | 1.181 | 2.06E-04 |
| MIMAT0030419 | hsa-miR-7844-5p | 1.181 | 2.28E-06 |
| MIMAT0005942 | hsa-miR-1288-3p | 1.180 | 2.24E-06 |
| MIMAT0019737 | hsa-miR-4664-5p | 1.179 | 2.57E-03 |
| MIMAT0017987 | hsa-miR-3610 | 1.179 | 8.76E-03 |
| MIMAT0019873 | hsa-miR-4742-3p | 1.179 | 1.16E-04 |
| MIMAT0018185 | hsa-miR-3911 | 1.178 | 9.36E-07 |
| MIMAT0026616 | hsa-miR-579-5p | 1.178 | 7.45E-05 |
| MIMAT0005930 | hsa-miR-1276 | 1.177 | 7.42E-06 |
| MIMAT0022842 | hsa-miR-98-3p | 1.177 | 2.03E-03 |
| MIMAT0003309 | hsa-miR-639 | 1.174 | 2.32E-05 |
| MIMAT0015003 | hsa-miR-3136-5p | 1.172 | 9.82E-04 |
| MIMAT0027032 | hsa-miR-500b-3p | 1.170 | 5.36E-03 |
| MIMAT0005875 | hsa-miR-548j-5p | 1.169 | 7.97E-04 |
| MIMAT0002838 | hsa-miR-525-5p | 1.166 | 5.02E-03 |
| MIMAT0027604 | hsa-miR-6852-5p | 1.166 | 1.33E-03 |
| MIMAT0019706 | hsa-miR-4645-3p | 1.166 | 2.56E-05 |
| MIMAT0004607 | hsa-miR-138-1-3p | 1.164 | 3.44E-04 |
| MIMAT0004488 | hsa-miR-15a-3p | 1.163 | 7.12E-05 |
| MIMAT0004785 | hsa-miR-545-5p | 1.163 | 1.17E-04 |
| MIMAT0022698 | hsa-miR-345-3p | 1.161 | 3.24E-05 |
| MIMAT0017991 | hsa-miR-3613-3p | 1.160 | 2.31E-03 |
| MIMAT0027454 | hsa-miR-6777-5p | 1.155 | 2.54E-03 |
| MIMAT0019935 | hsa-miR-4777-3p | 1.153 | 2.04E-04 |
| MIMAT0019871 | hsa-miR-4741 | 1.152 | 8.31E-07 |
| MIMAT0027541 | hsa-miR-6820-3p | 1.152 | 7.63E-05 |
| MIMAT0003306 | hsa-miR-636 | 1.152 | 3.68E-06 |
| MIMAT0020925 | hsa-miR-550a-3-5p | 1.149 | 9.21E-04 |
| MIMAT0019728 | hsa-miR-4660 | 1.149 | 7.67E-03 |
| MIMAT0003235 | hsa-miR-570-3p | 1.146 | 1.47E-03 |
| MIMAT0005892 | hsa-miR-1304-5p | 1.145 | 2.30E-03 |
| MIMAT0005924 | hsa-miR-1270 | 1.145 | 3.74E-03 |
| MIMAT0011777 | hsa-miR-2277-3p | 1.144 | 8.21E-04 |
| MIMAT0027497 | hsa-miR-6798-3p | 1.144 | 2.26E-05 |
| MIMAT0022709 | hsa-miR-652-5p | 1.142 | 2.54E-03 |
| MIMAT0005881 | hsa-miR-1291 | 1.141 | 2.78E-04 |
| MIMAT0018360 | hsa-miR-3944-3p | 1.141 | 3.04E-05 |
| MIMAT0022292 | hsa-miR-548au-3p | 1.139 | 1.98E-04 |
| MIMAT0025458 | hsa-miR-6501-5p | 1.139 | 7.41E-03 |
| MIMAT0027507 | hsa-miR-6803-3p | 1.139 | 9.88E-06 |
| MIMAT0027495 | hsa-miR-6797-3p | 1.138 | 6.82E-04 |
| MIMAT0019973 | hsa-miR-4797-3p | 1.137 | 1.84E-03 |
| MIMAT0019772 | hsa-miR-4685-3p | 1.137 | 7.01E-06 |
| MIMAT0004950 | hsa-miR-877-3p | 1.135 | 2.73E-03 |
| MIMAT0022500 | hsa-miR-5706 | 1.132 | 5.21E-05 |
| MIMAT0027654 | hsa-miR-6877-5p | 1.132 | 7.41E-03 |
| MIMAT0028220 | hsa-miR-7155-5p | 1.127 | 8.76E-04 |
| MIMAT0027517 | hsa-miR-6808-3p | 1.126 | 5.15E-05 |
| MIMAT0019712 | hsa-miR-4649-3p | 1.124 | 2.56E-05 |
| MIMAT0026623 | hsa-miR-627-3p | 1.122 | 1.10E-06 |
| MIMAT0027363 | hsa-miR-6731-5p | 1.121 | 2.60E-04 |
| MIMAT0019000 | hsa-miR-4473 | 1.121 | 4.27E-04 |
| MIMAT0007400 | hsa-miR-1538 | 1.121 | 1.32E-03 |
| MIMAT0019724 | hsa-miR-4657 | 1.118 | 8.17E-04 |
| MIMAT0003323 | hsa-miR-548d-3p | 1.117 | 4.87E-03 |
| MIMAT0019748 | hsa-miR-219b-3p | 1.116 | 9.15E-03 |
| MIMAT0027103 | hsa-miR-5699-5p | 1.110 | 1.22E-03 |
| MIMAT0014987 | hsa-miR-548s | 1.106 | 4.00E-03 |
| MIMAT0003246 | hsa-miR-581 | 1.106 | 2.21E-03 |
| MIMAT0015032 | hsa-miR-3158-3p | 1.105 | 1.61E-03 |
| MIMAT0003336 | hsa-miR-658 | 1.102 | 5.75E-03 |
| MIMAT0005941 | hsa-miR-1284 | 1.101 | 2.08E-04 |
| MIMAT0021022 | hsa-miR-5001-3p | 1.101 | 9.64E-03 |
| MIMAT0011163 | hsa-miR-548q | 1.095 | 4.10E-03 |
| MIMAT0018112 | hsa-miR-3684 | 1.095 | 2.68E-03 |
| MIMAT0019943 | hsa-miR-4781-3p | 1.093 | 2.11E-04 |
| MIMAT0022271 | hsa-miR-664b-5p | 1.092 | 2.44E-03 |
| MIMAT0003313 | hsa-miR-643 | 1.087 | 5.66E-03 |
| MIMAT0027505 | hsa-miR-6802-3p | 1.081 | 5.11E-03 |
| MIMAT0015031 | hsa-miR-3157-5p | 1.076 | 4.26E-03 |
| MIMAT0020959 | hsa-miR-4536-3p | -1.109 | 9.68E-03 |
| MIMAT0021117 | hsa-miR-5187-5p | -1.153 | 3.04E-03 |
| MIMAT0004674 | hsa-miR-30c-1-3p | -1.165 | 5.32E-03 |
| MIMAT0027513 | hsa-miR-6806-3p | -1.173 | 2.43E-03 |
| MIMAT0018204 | hsa-miR-676-3p | -1.184 | 3.40E-03 |
| MIMAT0005914 | hsa-miR-1262 | -1.189 | 3.31E-03 |
| MIMAT0022706 | hsa-miR-561-5p | -1.191 | 9.36E-04 |
| MIMAT0026618 | hsa-miR-585-5p | -1.192 | 3.66E-03 |
| MIMAT0000439 | hsa-miR-153-3p | -1.205 | 6.28E-03 |
| MIMAT0000083 | hsa-miR-26b-5p | -1.232 | 4.03E-03 |
| MIMAT0000734 | hsa-miR-380-5p | -1.238 | 6.80E-03 |
| MIMAT0000414 | hsa-let-7g-5p | -1.241 | 5.05E-04 |
| MIMAT0001629 | hsa-miR-329-3p | -1.246 | 6.04E-03 |
| MIMAT0000273 | hsa-miR-216a-5p | -1.248 | 4.66E-03 |
| MIMAT0000232 | hsa-miR-199a-3p | -1.257 | 5.52E-03 |
| MIMAT0004563 | hsa-miR-199b-3p | -1.258 | 5.41E-03 |
| MIMAT0004681 | hsa-miR-26a-2-3p | -1.262 | 8.94E-04 |
| MIMAT0003254 | hsa-miR-548b-3p | -1.267 | 1.44E-03 |
| MIMAT0004955 | hsa-miR-374b-5p | -1.271 | 6.98E-03 |
| MIMAT0022705 | hsa-miR-539-3p | -1.274 | 9.35E-04 |
| MIMAT0000090 | hsa-miR-32-5p | -1.278 | 4.05E-03 |
| MIMAT0000438 | hsa-miR-152-3p | -1.294 | 7.04E-03 |
| MIMAT0005878 | hsa-miR-1287-5p | -1.310 | 1.64E-03 |
| MIMAT0000737 | hsa-miR-382-5p | -1.321 | 7.79E-03 |
| MIMAT0004592 | hsa-miR-125b-1-3p | -1.321 | 4.08E-04 |
| MIMAT0004688 | hsa-miR-374a-3p | -1.326 | 2.46E-05 |
| MIMAT0000431 | hsa-miR-140-5p | -1.334 | 1.11E-05 |
| MIMAT0010214 | hsa-miR-151b | -1.339 | 7.61E-05 |
| MIMAT0003879 | hsa-miR-758-3p | -1.353 | 2.10E-03 |
| MIMAT0000062 | hsa-let-7a-5p | -1.354 | 8.06E-05 |
| MIMAT0004615 | hsa-miR-195-3p | -1.360 | 1.04E-04 |
| MIMAT0000730 | hsa-miR-377-3p | -1.362 | 1.52E-03 |
| MIMAT0003266 | hsa-miR-598-3p | -1.364 | 1.68E-04 |
| MIMAT0000245 | hsa-miR-30d-5p | -1.374 | 3.99E-05 |
| MIMAT0000437 | hsa-miR-145-5p | -1.386 | 2.65E-03 |
| MIMAT0003386 | hsa-miR-376a-5p | -1.393 | 2.59E-03 |
| MIMAT0004515 | hsa-miR-29b-2-5p | -1.394 | 8.15E-04 |
| MIMAT0004683 | hsa-miR-362-3p | -1.395 | 1.35E-05 |
| MIMAT0004551 | hsa-miR-30d-3p | -1.398 | 6.23E-11 |
| MIMAT0000272 | hsa-miR-215-5p | -1.398 | 3.64E-04 |
| MIMAT0004809 | hsa-miR-628-5p | -1.403 | 4.46E-04 |
| MIMAT0000447 | hsa-miR-134-5p | -1.411 | 1.27E-03 |
| MIMAT0000067 | hsa-let-7f-5p | -1.413 | 1.54E-04 |
| MIMAT0030019 | hsa-miR-7704 | -1.423 | 5.00E-03 |
| MIMAT0019071 | hsa-miR-4532 | -1.430 | 7.19E-04 |
| MIMAT0000418 | hsa-miR-23b-3p | -1.436 | 7.29E-06 |
| MIMAT0003161 | hsa-miR-493-3p | -1.445 | 1.75E-03 |
| MIMAT0003332 | hsa-miR-656-3p | -1.454 | 4.74E-05 |
| MIMAT0005948 | hsa-miR-664a-5p | -1.454 | 1.00E-08 |
| MIMAT0005920 | hsa-miR-1266-5p | -1.473 | 4.13E-04 |
| MIMAT0004604 | hsa-miR-127-5p | -1.497 | 3.58E-04 |
| MIMAT0000420 | hsa-miR-30b-5p | -1.522 | 5.65E-04 |
| MIMAT0000707 | hsa-miR-363-3p | -1.578 | 1.73E-05 |
| MIMAT0005899 | hsa-miR-1247-5p | -1.597 | 2.87E-03 |
| MIMAT0000727 | hsa-miR-374a-5p | -1.604 | 1.37E-09 |
| MIMAT0002818 | hsa-miR-496 | -1.624 | 9.31E-05 |
| MIMAT0000729 | hsa-miR-376a-3p | -1.642 | 3.27E-04 |
| MIMAT0004692 | hsa-miR-340-5p | -1.646 | 3.28E-06 |
| MIMAT0000419 | hsa-miR-27b-3p | -1.705 | 1.13E-06 |
| MIMAT0000094 | hsa-miR-95-3p | -1.708 | 7.15E-03 |
| MIMAT0003328 | hsa-miR-653-5p | -1.731 | 1.13E-05 |
| MIMAT0022862 | hsa-miR-381-5p | -1.757 | 7.86E-03 |
| MIMAT0026472 | hsa-let-7c-3p | -1.771 | 1.15E-06 |
| MIMAT0000452 | hsa-miR-154-5p | -1.786 | 2.37E-06 |
| MIMAT0006789 | hsa-miR-1468-5p | -1.827 | 3.61E-09 |
| MIMAT0004600 | hsa-miR-144-5p | -1.830 | 5.19E-03 |
| MIMAT0004673 | hsa-miR-29c-5p | -1.842 | 2.68E-06 |
| MIMAT0005909 | hsa-miR-1258 | -1.845 | 7.52E-07 |
| MIMAT0000763 | hsa-miR-338-3p | -1.847 | 8.80E-05 |
| MIMAT0000444 | hsa-miR-126-5p | -1.861 | 1.14E-09 |
| MIMAT0002820 | hsa-miR-497-5p | -1.880 | 8.35E-12 |
| MIMAT0004902 | hsa-miR-891a-5p | -1.884 | 4.32E-06 |
| MIMAT0004690 | hsa-miR-379-3p | -1.886 | 1.39E-05 |
| MIMAT0004813 | hsa-miR-411-3p | -1.887 | 2.02E-04 |
| MIMAT0000458 | hsa-miR-190a-5p | -1.895 | 3.22E-08 |
| MIMAT0004597 | hsa-miR-140-3p | -1.914 | 1.29E-13 |
| MIMAT0004921 | hsa-miR-889-3p | -1.949 | 3.32E-08 |
| MIMAT0004947 | hsa-miR-885-5p | -1.957 | 1.84E-03 |
| MIMAT0000263 | hsa-miR-199b-5p | -1.982 | 1.39E-06 |
| MIMAT0000423 | hsa-miR-125b-5p | -1.987 | 7.72E-13 |
| MIMAT0003331 | hsa-miR-655-3p | -1.989 | 3.76E-05 |
| MIMAT0000275 | hsa-miR-218-5p | -1.999 | 2.90E-09 |
| MIMAT0026483 | hsa-miR-370-5p | -2.015 | 1.02E-09 |
| MIMAT0022929 | hsa-miR-758-5p | -2.025 | 1.90E-06 |
| MIMAT0004549 | hsa-miR-148a-5p | -2.028 | 5.11E-04 |
| MIMAT0000448 | hsa-miR-136-5p | -2.069 | 8.49E-05 |
| MIMAT0000693 | hsa-miR-30e-3p | -2.077 | 4.62E-11 |
| MIMAT0001631 | hsa-miR-451a | -2.146 | 1.08E-03 |
| MIMAT0002814 | hsa-miR-432-5p | -2.155 | 2.44E-07 |
| MIMAT0000082 | hsa-miR-26a-5p | -2.173 | 6.35E-12 |
| MIMAT0000765 | hsa-miR-335-5p | -2.185 | 1.01E-08 |
| MIMAT0004757 | hsa-miR-431-3p | -2.190 | 5.59E-05 |
| MIMAT0000692 | hsa-miR-30e-5p | -2.192 | 3.31E-11 |
| MIMAT0003180 | hsa-miR-487b-3p | -2.220 | 5.46E-08 |
| MIMAT0018926 | hsa-miR-378d | -2.265 | 1.61E-05 |
| MIMAT0000274 | hsa-miR-217-5p | -2.266 | 1.89E-06 |
| MIMAT0004513 | hsa-miR-101-5p | -2.299 | 1.07E-10 |
| MIMAT0000086 | hsa-miR-29a-3p | -2.309 | 2.75E-14 |
| MIMAT0000250 | hsa-miR-139-5p | -2.324 | 6.73E-07 |
| MIMAT0004701 | hsa-miR-338-5p | -2.329 | 1.08E-09 |
| MIMAT0000721 | hsa-miR-369-3p | -2.329 | 5.55E-08 |
| MIMAT0002883 | hsa-miR-514a-3p | -2.336 | 6.43E-04 |
| MIMAT0004601 | hsa-miR-145-3p | -2.337 | 3.02E-13 |
| MIMAT0004511 | hsa-miR-99a-3p | -2.338 | 2.20E-11 |
| MIMAT0001621 | hsa-miR-369-5p | -2.409 | 8.33E-09 |
| MIMAT0004689 | hsa-miR-377-5p | -2.435 | 4.61E-07 |
| MIMAT0002880 | hsa-miR-508-3p | -2.548 | 1.64E-04 |
| MIMAT0000436 | hsa-miR-144-3p | -2.552 | 2.76E-05 |
| MIMAT0000731 | hsa-miR-378a-5p | -2.584 | 2.69E-05 |
| MIMAT0000435 | hsa-miR-143-3p | -2.587 | 3.86E-07 |
| MIMAT0002817 | hsa-miR-495-3p | -2.600 | 7.34E-07 |
| MIMAT0026478 | hsa-miR-133a-5p | -2.608 | 2.30E-03 |
| MIMAT0000446 | hsa-miR-127-3p | -2.885 | 2.97E-09 |
| MIMAT0004960 | hsa-miR-208b-3p | -2.977 | 2.91E-04 |
| MIMAT0004814 | hsa-miR-654-3p | -3.031 | 1.43E-09 |
| MIMAT0000754 | hsa-miR-337-3p | -3.073 | 1.01E-08 |
| MIMAT0002177 | hsa-miR-486-5p | -3.099 | 1.77E-05 |
| MIMAT0000720 | hsa-miR-376c-3p | -3.146 | 1.07E-08 |
| MIMAT0025477 | hsa-miR-6510-3p | -3.149 | 4.10E-05 |
| MIMAT0000732 | hsa-miR-378a-3p | -3.195 | 2.31E-07 |
| MIMAT0000098 | hsa-miR-100-5p | -3.215 | 1.59E-19 |
| **MIMAT0002870** | **hsa-miR-499a-5p** | **-3.296** | **3.76E-05** |
| **MIMAT0000733** | **hsa-miR-379-5p** | **-3.298** | **1.29E-10** |
| **MIMAT0002890** | **hsa-miR-299-5p** | **-3.504** | **8.97E-07** |
| **MIMAT0000461** | **hsa-miR-195-5p** | **-3.510** | **7.79E-14** |
| **MIMAT0022721** | **hsa-miR-1247-3p** | **-3.553** | **3.40E-07** |
| **MIMAT0016847** | **hsa-miR-378c** | **-3.670** | **4.61E-08** |
| **MIMAT0002171** | **hsa-miR-410-3p** | **-3.684** | **9.33E-12** |
| **MIMAT0004603** | **hsa-miR-125b-2-3p** | **-3.694** | **1.52E-18** |
| **MIMAT0004606** | **hsa-miR-136-3p** | **-3.797** | **1.08E-12** |
| **MIMAT0004550** | **hsa-miR-30c-2-3p** | **-3.881** | **1.03E-12** |
| **MIMAT0004552** | **hsa-miR-139-3p** | **-3.937** | **3.02E-14** |
| **MIMAT0000099** | **hsa-miR-101-3p** | **-4.017** | **3.64E-23** |
| **MIMAT0000087** | **hsa-miR-30a-5p** | **-4.132** | **6.93E-14** |
| **MIMAT0003329** | **hsa-miR-411-5p** | **-4.160** | **2.03E-10** |
| **MIMAT0000265** | **hsa-miR-204-5p** | **-4.519** | **1.28E-17** |
| **MIMAT0000681** | **hsa-miR-29c-3p** | **-4.539** | **5.24E-17** |
| **MIMAT0000064** | **hsa-let-7c-5p** | **-4.674** | **3.68E-22** |
| **MIMAT0000462** | **hsa-miR-206** | **-5.228** | **4.62E-03** |
| **MIMAT0000736** | **hsa-miR-381-3p** | **-5.293** | **5.06E-08** |
| **MIMAT0000770** | **hsa-miR-133b** | **-5.580** | **3.66E-04** |
| **MIMAT0000088** | **hsa-miR-30a-3p** | **-5.696** | **2.66E-13** |
| **MIMAT0000097** | **hsa-miR-99a-5p** | **-5.746** | **1.85E-27** |
| **MIMAT0000427** | **hsa-miR-133a-3p** | **-7.055** | **2.93E-04** |
| **MIMAT0000416** | **hsa-miR-1-3p** | **-10.663** | **8.80E-06** |
| **MIMAT0000728** | **hsa-miR-375-3p** | **-18.183** | **1.33E-11** |

In bold the 50 selected miRNAs;*p-values were calculated by Student’s t-test
